# Non-coding *cis*-regulatory variants in *HK1* cause congenital hyperinsulinism with variable disease severity

**DOI:** 10.1186/s13073-025-01440-w

**Published:** 2025-03-03

**Authors:** Jasmin J. Bennett, Cécile Saint-Martin, Bianca Neumann, Jonna M. E. Männistö, Jayne A. L. Houghton, Susann Empting, Matthew B. Johnson, Thomas W. Laver, Jonathan M. Locke, Benjamin Spurrier, Matthew N. Wakeling, Indraneel Banerjee, Antonia Dastamani, Hüseyin Demirbilek, John Mitchell, Markus Stange, International Congenital Hyperinsulinism Consortium, Marie-Thérèse Abi Warde, Mehta Amrita, Romy Aravena, Alina Arion, Navoda Atapattu, Ivo Barić, Jérôme Bertherat, Esra Bilici, Juliette Bouchereau, Karine Braun, Marie-Neige Campas-Lebecque, Mireille Castanet, Catie Cessans, Louise S. Conwell, Preeti Dabadghao, Archana Dayal Arya, Pascale de Lonlay, Liat de Vries, Céline Droumaguet, Noémie Faure-Galon, Olivier Gilly, Alice Goldenberg, Anne-Sophie Guemann, Anne-Marie Guerrot, Julie Harvengt, Samar S. Hassan, Saw Shi Hui, Khadija Nuzhat Humayun, M. Ibrahim, Vandana Jain, Dhivyalakshmi Jeevarathnam, Kah Yin Loke, Vaman Khadilkar, I. P. S. Kochar, Abhishek Kulkarni, Aniket Kumbhojkar, Delphine Lamireau, Floris Levy-Khademi, Catarina Limbert, Martin Lindner, Catherine Lombard, François Maillot, Karine Mention, Verónica Mericq, Zainaba Mohamed, Coline Mornet, Philip Murray, Alexandre Naccache, Lusine V. Navasardyan, Kristen Neville, Ramona Nicolescu, Marc Nicolino, Elisa Nishimura-Meguro, Nattakarn Numsriskulrat, Sinead O’sullivan, Yasmine Ouarezki, Armelle Pambou, Florence Petit, V. P. Praveen, Mélanie Priou-Guesdon, Stoeva Radka, Birgit Rami-Merhar, Sudha Rao, Yves Reznik, Laurence Rulquin, Maria Salomon Estebanez, Isabelle Souto, Antoine Tabarin, Ana Tangari, Sara Van Aken, Charles Verge, Hélène Vinolas, Christel Voinot, Robert Wagner, Jan Walker, Esko Wiltshire, Klaus Mohnike, Jean-Baptiste Arnoux, Nick D. L. Owens, Martin Zenker, Christine Bellanné-Chantelot, Sarah E. Flanagan

**Affiliations:** 1 Department of Clinical and Biomedical Sciences, Faculty of Health and Life Sciences, University of Exeter, Exeter, EX2 5DW UK; 2 Department of Medical Genetics, AP-HP Sorbonne University, Pitié-Salpêtrière Hospital, 75013 Paris, France; 3 Institute of Human Genetics, University Hospital, Otto-Von-Guericke University Magdeburg, Leipziger Str. 44, Magdeburg, 39120 Germany; 4 Kuopio Pediatric Research Unit (KuPRU), University of Eastern Finland, Kuopio, 70029 Finland; 5 Exeter Genomics Laboratory, Royal Devon University Healthcare NHS Foundation Trust, Exeter, EX2 5DW UK; 6 Children’s University Hospital, Otto-Von-Guericke University Magdeburg, Leipziger Str. 44, Magdeburg, 39120 Germany; 7 Department of Paediatric Endocrinology, Royal Manchester Children’s Hospital, Manchester, M13 9WL UK; 8 Endocrinology Department, Great Ormond Street Hospital for Children, London, WC1N 3JH UK; 9 Faculty of Medicine, Department of Pediatric Endocrinology, Hacettepe University, Ankara, Turkey; 10 Pediatric Endocrinology and Biochemical Genetics, Human Genetics and Pediatrics, Montreal Children’s Hospital-McGill University, McGill University, Montreal, Canada; 11 Department of Pediatrics, University Hospital Halle, Ernst Grube Str. 40, Halle, 06120 Germany; 12 Reference Center for Inherited Metabolic Diseases, Necker-Enfants-Malades University Hospital, APHP, Imagine Institute, G2M, MetabERN, Paris Cité University, Paris, 75015 France

**Keywords:** Congenital hyperinsulinism, Non-coding, Hexokinase 1, Monogenic disease, Variable penetrance

## Abstract

**Background:**

We recently reported non-coding variants in a *cis*-regulatory element of the beta-cell disallowed gene hexokinase 1 (*HK1*) as a novel cause of congenital hyperinsulinism. These variants lead to a loss of repression of HK1 in pancreatic beta-cells, causing insulin secretion during hypoglycaemia. In this study, we aimed to determine the prevalence, genetics, and phenotype of *HK1*-hyperinsulinism by screening a large international cohort of patients living with the condition.

**Methods:**

We screened the *HK1 cis*-regulatory region in 1761 probands with hyperinsulinism of unknown aetiology who had been referred to one of three large European genomics laboratories.

**Results:**

We identified a *HK1* variant in 89/1761 probands (5%) and 63 family members. Within the Exeter HI cohort, these variants accounted for 2.8% of all positive genetic diagnoses (*n* = 54/1913) establishing this as an important cause of HI. Individuals with a disease-causing variant were diagnosed with hyperinsulinism between birth and 26 years (median: 7 days) with variable response to treatment; 80% were medically managed and 20% underwent pancreatic surgery due to poor response to medical therapy. Glycaemic outcomes varied from spontaneous remission to hypoglycaemia persisting into adulthood. Eight probands had inherited the variant from a parent not reported to have hyperinsulinism (median current age: 39 years), confirming variable penetrance. Two of the 23 novel *HK1* variants allowed us to extend the minimal *cis*-regulatory region from 42 to 46 bp.

**Conclusions:**

Non-coding variants within the *HK1 cis*-regulatory region cause hyperinsulinism of variable severity ranging from neonatal-onset, treatment-resistant disease to being asymptomatic into adulthood. Discovering variants in 89 families confirms *HK1* as a major cause of hyperinsulinism and highlights the important role of the non-coding genome in human monogenic disease.

**Supplementary Information:**

The online version contains supplementary material available at 10.1186/s13073-025-01440-w.

## Background

The *HK1 *gene encodes the glycolytic enzyme hexokinase 1 which is expressed across all human tissues except for the pancreas and liver [1]. Silencing of *HK1 *in pancreatic beta-cells is essential for appropriate glucose sensing and glucose-induced insulin secretion which instead is controlled by glucokinase (GCK or hexokinase 4), an enzyme with up to fivefold lower affinity for glucose than HK1 [2]. Silencing of *HK1* in favour of *GCK* therefore prevents insulin secretion at low blood glucose levels.

We recently demonstrated that large de novo deletions, single nucleotide variants (SNVs) and indels affecting a 42 bp intronic region of *HK1 *can cause congenital hyperinsulinism (HI), a condition defined by the inadequate suppression of plasma insulin during hypoglycaemia [3, 4]. These dominantly acting, non-coding variants affected a *cis*-regulatory element bound by beta-cell transcription factors. Analysis of affected pancreatic tissue demonstrated that *HK1 *silencing was lost within the beta-cells, which lowered the set point for glucose metabolism resulting in severe HI. The clinical severity of this genetic form of HI was demonstrated by presentation in early infancy, poor response to the drug diazoxide, with doses exceeding 10 mg/kg/day in eight individuals, and the need for a pancreatectomy to control hypoglycaemia in five children [3]. These findings provided a rare example of highly penetrant deleterious variants affecting a *cis*-regulatory region, highlighting the importance of the non-coding genome in the aetiology of human monogenic disease [5, 6].

In the previous study, *HK1 *variants were identified in 9% of probands (14/162 screened) [3]. These individuals were selected for testing as they had a severe phenotype suggestive of a monogenic aetiology. Consequently, the original study could not establish whether phenotypic variability exists within *HK1*-HI as described in other genetic forms of HI. For example, individuals with activating *GCK* variants (*GCK*-HI) can present with HI at birth which responds poorly to diazoxide [7], or present outside of infancy and show good response to medical therapy [8–10]. At the mildest end of the spectrum are family members of affected probands who carry the causative *GCK *variant but remain clinically unaffected into mid-adulthood [11, 12].

There is evidence that phenotypic variability within *HK1*-HI exists. In 2013, 9 years prior to the discovery by Wakeling et al., Henquin et al. demonstrated a role for the glycolytic enzyme in the pathophysiology of HI by detecting aberrant expression of *HK1 *in pancreatic tissue from five children with HI of unknown genetic cause [3, 13]. Age at diagnosis ranged from the neonatal period to 6 months with two individuals receiving high doses of diazoxide (15–18 mg/kg/day) suggesting non-responsiveness to the drug [13, 14]. In a separate study, linkage analysis identified an 8.2-Mb region on chromosome 10q21–22, encompassing *HK1*, in a large pedigree with dominantly inherited HI that responded to diazoxide [15]. Sequencing analysis subsequently detected three intronic *HK1* variants, one of which (GRCh37:Chr10:g.71,108,666dup) was within the regulatory region later described by Wakeling et al. [3]. In that family, HI was diagnosed between the ages of 3 and 17 months in all but one individual who was diagnosed with hypoglycaemia at the age of 89 years. Five unaffected obligate heterozygotes were also identified. More recently, a *HK1 *variant of uncertain clinical significance was reported in an individual from Norway with diazoxide-responsive HI diagnosed at the age of 5.5 months. The child had inherited the variant from its mother whose glycaemic status was not known [16].

In this current study, we investigate the genetics and prevalence of *HK1*-HI, and explore the phenotypic spectrum associated with this condition. Through these studies, we firmly establish that *HK1 cis*-regulatory variants are a common cause of HI that can exhibit phenotypic variability between and within families.

## Methods

### Cohort studied

We studied 1761 probands referred by their clinician for HI genetic testing over a period of 20 years to three European genomic centres: Exeter, UK (*n* = 1090; 2004–2023), Paris, France (*n* = 486; 2003–2023), and Magdeburg, Germany (*n*= 185; 2012–2022). All probands had received a clinical diagnosis of HI as defined by the finding of increased insulin action and/or inadequate suppression of plasma insulin during spontaneous or fasting-induced hypoglycaemia [17]. All individuals were recruited into the study by their clinicians.

The Exeter unsolved cohort (*n* = 1090) consisted of UK (*n* = 370) and internationally (*n* = 720) referred patients. Of these, 162 individuals were included in the study by Wakeling et al. [3]. Disease-causing variants in at least 12 known HI genes had been excluded in all individuals by targeted next-generation sequencing (tNGS) (*ABCC8*, *CACNA1D*, *GCK*, *GLUD1*, *HADH*, *HNF1A*, *HNF4A*, *INSR*, *KCNJ11*, *PMM2*,* SLC16A1*, and *TRMT10A*) [18]. To compare the frequency of *HK1* variants to other known genetic causes of HI, we collated variant data on genetically solved individuals from the Exeter cohort (*n* = 1859) (Additional file 1: Fig. S1).

In all 486 individuals referred to Paris, disease-causing variants in the *ABCC8* and *KCNJ11* genes had been excluded by Sanger sequencing (*n* = 131) or tNGS (*n* = 355). Sanger sequencing or tNGS had also excluded disease-causing variants in additional HI genes in a subset of individuals; *GLUD1* (*n* = 207), *GCK* (*n* = 389), *HADH* (*n* = 205), *HNF4A* (*n* = 421), and *HNF1A* (*n* = 437).

The Magdeburg cohort included 185 individuals in whom disease-causing variants in the *ABCC8* and *KCNJ11* genes had been excluded by Sanger sequencing or tNGS. In 142 of these individuals, disease-causing variants were excluded by tNGS in a subset of the following genes: *GLUD1*, *GCK*, *HNF4A*, *HNF1A*, *HADH*, *SLC16A1*, *INSR*, and *TRMT10A*.

The study was conducted in accordance with the Declaration of Helsinki principles with informed written consent obtained from the parents of all participants included in this study. The study was approved by the Wales Research Ethics Committee 5 (22/WA/0268), with participants recruited to the Genetic Beta Cell Research Bank (IRAS: 316,050), and the ethics committee of the Otto von Guericke University at the Medical Faculty and at the University Hospital Magdeburg A.ö.R, Leipziger Str. 44, 39,120 Magdeburg (Ethics Board vote (110/04)). Appropriate consent in accordance with national regulations for diagnostic genetic testing and the scientific use of anonymized/pseudonymized genetic and clinical data was obtained.

### *HK1* sequencing

Sequencing analysis of the *HK1 cis*-regulatory element was performed on leukocyte DNA from 1761 probands. The minimum region screened in all individuals was GRCh37:Chr10:71,108,536–71,108,807. This was performed using whole genome sequencing (WGS) (135 individuals as previously described [3]) or Sanger sequencing (1626 individuals). Details of PCR primers and sequencing analysis pipelines are provided in Additional file 1: Table S1. Variants that were absent from gnomAD v3 were considered for follow-up [19]. When a mosaic variant was identified, allele fractions were calculated from Sanger sequencing data using the standardised allele ratio in Mutation Surveyor version 3.24 (SoftGenetics, Rouen, France) [20], or by read-depth analysis from WGS data [21].

### Deletion screening

When sequencing analysis did not identify a rare variant (*n* = 1689), detection of common heterozygous variants within the sequencing data were used to exclude a large deletion over the critical regulatory region (*n*= 556/1689; 33%). The common variants used were: rs7093863 (minor allele frequency (MAF): 0.14) and/or rs7094214 (MAF: 0.24) in Exeter, Paris and Magdeburg cohorts or rs151188129 (MAF: 0.003) and rs12257925 (MAF: 0.009) in the Paris cohort only [19]. Deletion analysis was performed on the 1133 remaining probands.

For the Exeter cohort (*n* = 679), deletion analysis involved read depth analysis of WGS data (*n* = 90), droplet digital PCR (ddPCR) (*n* = 17), or quantitative PCR (qPCR) (*n*= 572). For qPCR, quadruplet reactions (5 μl) containing a custom TaqMan Copy Number Assay (Applied Biosystems, Waltham, MA, USA, Additional file 1: Table S2) and genomic DNA (20 ng) were amplified. Fluorescence was detected using a QuantStudio 12 K Flex and data analysed using CopyCaller v2.1 analysis software (Thermo Fisher Scientific, Waltham, MA, USA). Details of the WGS analysis and ddPCR have been reported previously [3, 21].

For the Paris cohort (*n* = 314), deletion analysis was performed by qPCR using a SYBR Green assay. Briefly, triplicate qPCR reactions were carried out on an ABI7500 Real-Time PCR System with data analysed using SDS software v2.4 (Applied Biosystems, Waltham, MA, USA) (Additional file 1: Table S2).

For the Magdeburg cohort (*n* = 140), deletion analysis was performed by multiplex ligation-dependent probe amplification (MLPA). Briefly, custom probes spanning the region were designed and used in a reaction mix with a P300 reference MLPA kit as per the manufacturer’s instructions (MRC Holland, The Netherlands). Following analysis on an ABI 3500xl genetic analyser (Life Technologies GmbH, Darmstadt, Germany) obtained data were analysed using the Sequence Pilot software v.5.3.4 (JSI medical systems, Ettenheim, Germany). Probe sequences are provided in Additional file 1: Table S2.

### Family member testing

When available, samples from parents and additional affected family members of individuals with a *HK1* variant underwent sequencing or deletion analysis. When a de novo variant was identified, parental relationships were confirmed using WGS trio data or by genome-wide microsatellite analysis (PowerPlex, Promega, Southampton, UK).

### Variant classification

Variants were assessed according to the guidelines for interpretation of variants in the non-coding genome, based on the ACMG variant classification guidelines, and the ACGS Best Practice Guidelines for Variant Classification in Rare Disease [22–24].

### Phenotypic analysis

Clinical data were provided for all cases at referral using a laboratory request form with follow-up information obtained by case-note review where possible. Birth weight *Z*-scores were calculated using WHO standards, accessed through the Zanthro package in Stata 16 (StataCorp, Texas, USA) [25].

## Results

We identified a dominant *HK1* variant in 89 probands with HI, representing an overall prevalence of 5% (89/1761) in genetically unsolved individuals. The pick-up rates were similar across the three centres: Exeter: 54/1090 (5%), Paris: 29/486 (6%), Magdeburg: 6/185 (3%). Within the Exeter cohort, this represented an overall pick-up rate of 1.8% (*n* = 54/2949) and 2.8% of the positive genetic diagnoses (*n* = 54/1913) (Additional file 1: Fig. S1).

**Fig. 1:**
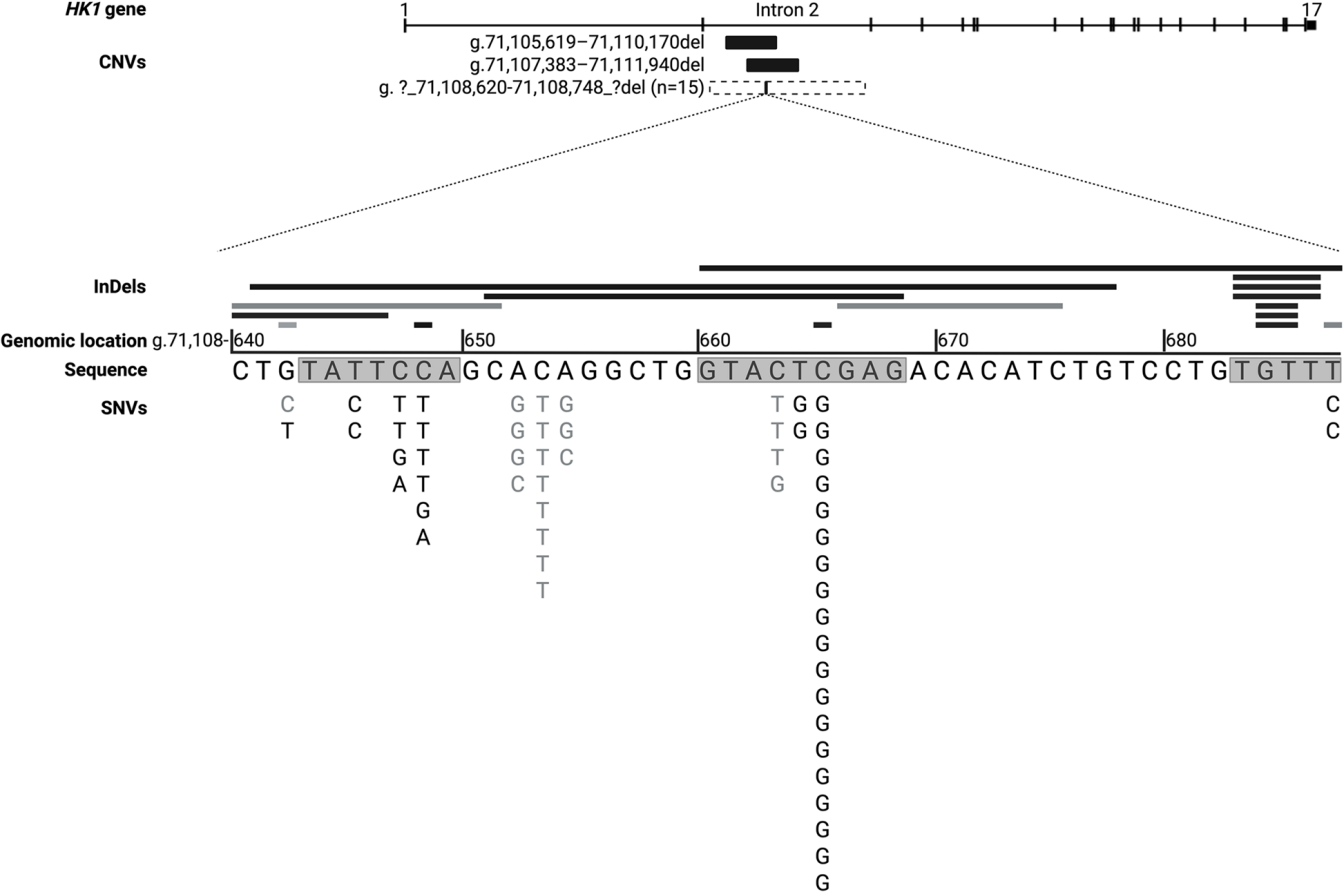
Schematic representation of the location of *HK1* variants identified in probands with congenital hyperinsulinism. The ubiquitously expressed isoform (ENST00000359426) is depicted. The position of copy number variants (CNVs) within intron 2 is shown below the *HK1 *gene. The breakpoints of the large deletions have been defined in two cases, the remaining 15 are known to extend beyond the sequenced region but not into the coding sequence as depicted by the dashed line. The horizontal bars depict indels. Only bases within the indel that are affecting the minimal regulatory region are shown (Chr10:71,108,642–71108687). The g.71,108,688–71,108,691del has been named according to HGVS nomenclature, this variant deletes a TGTT repeated sequence that starts at g.71,108,683. Directly below the indels is the reference sequence. The greyed boxes around the nucleotide bases of the genomic sequence indicate the previously defined predicted transcription factor binding sites [3]. Single nucleotide variants (SNVs) are listed, according to position, below the genomic sequence. Black lines/text indicate variants classified as pathogenic or likely pathogenic and grey lines/text indicate those classified as variants of uncertain significance according to the current classification guidelines [22–24]

Thirty-two different variants were detected, of which 23 (72%) were novel [3, 16]. Nineteen of the variants were SNVs (56/89 probands), 12 were indels (16/89 probands), and 17 probands had a large deletion encompassing the critical region (Fig. 1).

### Inheritance of *HK1* variants

**Fig. 2:**
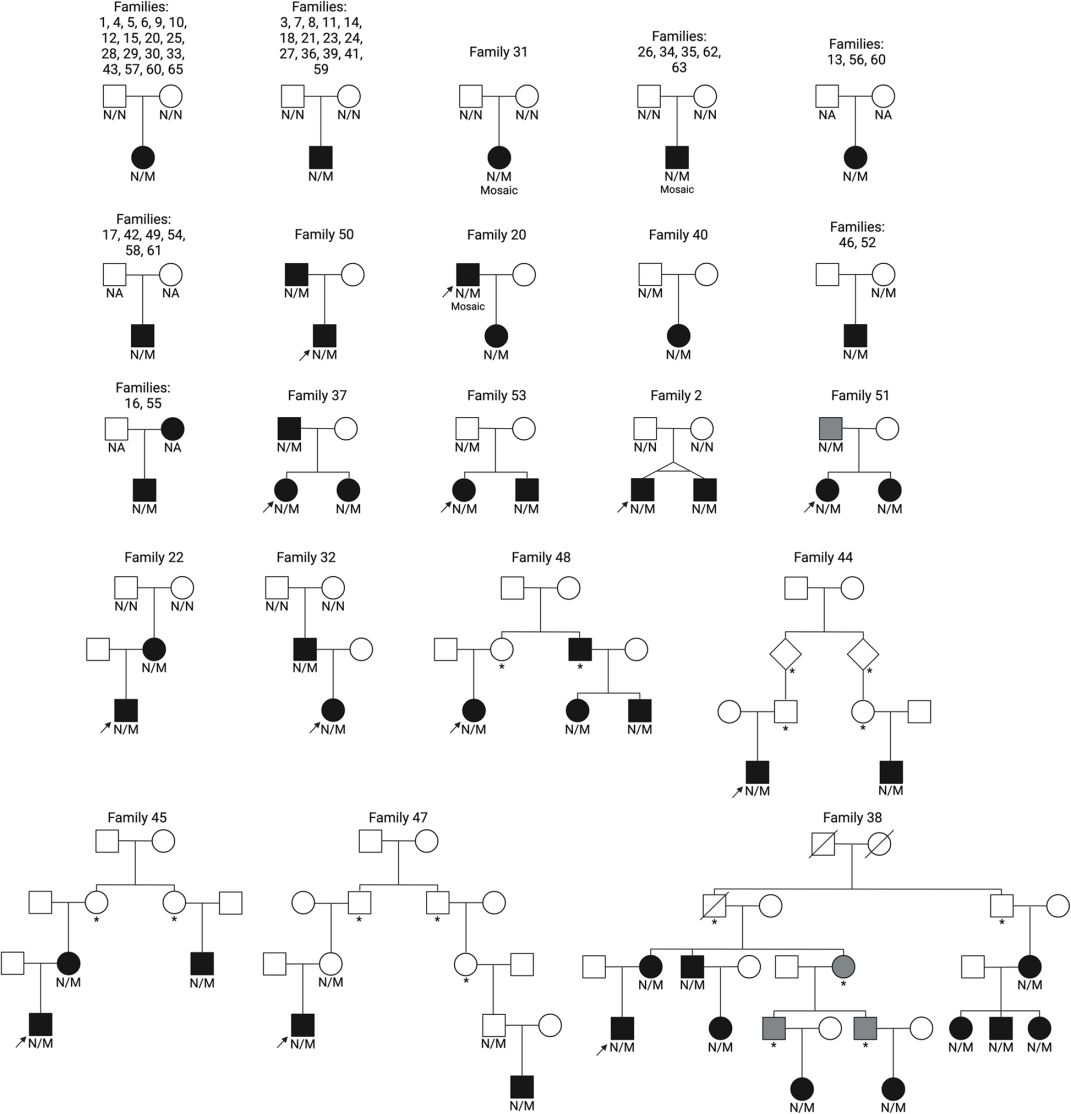
Pedigrees depicting inheritance of pathogenic and likely pathogenic *HK1* variants (*n* = 65 families). Squares, males; circles, females; diamonds, unknown sex; filled black symbols, clinical diagnosis of hyperinsulinism; filled grey symbols, anecdotal evidence of hypoglycaemia; diagonal line through symbol, deceased; M, *HK1* variant; N, no variant; *, obligate heterozygote; NA, DNA not available. The arrow indicates the proband in larger pedigrees

Forty of the 89 probands (45%) had a de novo variant. In seven of these, the variant was mosaic (~ 12–34% in leukocyte DNA). Eight probands (9%) had inherited the variant from a parent with HI, and 18 (20%) had inherited the variant from a parent not reported to have HI (including 2 obligate heterozygotes). For the remaining 23 (26%) probands, inheritance could not be established. The familial *HK1* variant was also present in 37 additional family members (including 14 obligate heterozygotes) of 14 probands (Fig. 2, Additional file 1: Fig. S2).

### *HK1* variant classification

There was sufficient evidence to classify 20 of the 32 different variants as pathogenic or likely pathogenic [22, 23]. This included as a minimum the absence of the variant in multiple population datasets (PM2_moderate) [19, 26–30], and the finding of a de novo variant in at least one individual with HI (PS2_strong) (Fig. 1, Additional file 1: Table S3). These pathogenic and likely pathogenic variants were identified in 112 individuals (65 probands, 31 family members, and 16 obligate heterozygotes) (Fig. 2).

The remaining 12 variants were classified as variants of uncertain significance (VUS) [22, 23]. Although insufficient, there was evidence to support their causality including absence in multiple population datasets (PM2_moderate) [19, 26–29] and affecting a base that was disrupted in two or more unrelated individuals with HI (PS4_moderate). Five of the variants were also predicted to disrupt a binding motif of a transcription factor, the perturbation of which is repeatedly shown to be pathogenic (PM1_supporting) (Fig. 1, Additional file 1: Table S3) [3]. These VUSs were identified in 40 individuals (24 probands and 16 family members) (Additional file 1: Fig. S2).

### Clinical characteristics of individuals with a pathogenic or likely pathogenic *HK1* variant

HI had been clinically diagnosed in 80% (90/112) of individuals with a pathogenic or likely pathogenic *HK1* variant (65 probands and 25 family members, 17 reported previously) [3]. The median age at diagnosis was 7 days (IQR: birth–9 months) with 54% diagnosed in the neonatal period (≤ 28 days of age), 25% diagnosed in infancy (1 month–12 months), 16% diagnosed in childhood (1 year–17 years), and 4% diagnosed in adulthood (≥ 18 years). Large for gestational age birthweights (> 2 SDS) were reported in 16/66 (24%) individuals where data was available. Of these, all had neonatal onset HI supporting increased insulin secretion in utero [31]. No difference in the median age at diagnosis of HI or birth weight Z-score was observed between probands and affected family members (*P* = 0.06 and *P* = 0.7, respectively, Mann–Whitney *U*) (Table 1, Additional file 1: Table S4).

**Table 1:** Summary of clinical data for 90 individuals with a clinical diagnosis of congenital hyperinsulinism and a pathogenic or likely pathogenic variant within the *HK1* regulatory region

	**Probands *n* = 65**	**Affected family members *n* = 25**	**Combined *n* = 90**
Median age at follow-up in years (range) (*n*)	5 (0.2–66) (60)	9 (2–43) (21)	6 (0.2–66) (81)
Female sex, % (*n*)	45% (29/65)	52% (13/25)	47% (42/90)
Median age at diagnosis, [IQR] (*n*)	3 days [birth–6 months]* (65/65)	8 months [35 days–2 years]* (14/25)	7 days [birth–8.5 months] (79/90)
Neonatal onset HI, % (*n*)	60% (39/65)	29% (4/14)	54% (43/79)
Infancy-onset HI, % (*n*)	23% (15/65)	36% (5/14)	25% (20/79)
Childhood-onset HI, % (*n*)	15% (10/65)	21% (3/14)	16% (13/79)
Adult-onset HI, % (*n*)	2% (1/65)	14% (2/14)	4% (3/79)
Median glucose at presentation, mmol/L (paired insulin, pmol/L) (*n*)	1.45 (150) (48/65)	1.9 (58.5) (6/25)	1.5 (118) (54/90)
Median birth weight *Z*-score, [IQR] (*n*)	0.47 [− 0.12–1.99]* (57/65)	0.58 [0.12–0.79]* (9/25)	0.48 [− 0.12–1.97] (66/90)
Medical management, % (*n*)	81% (50/62)	77% (10/13)	80% (60/75)
Diazoxide only, % (*n*)	78% (39/50)	80% (8/10)	77% (46/60)
Diazoxide + somatostatin receptor analogue combined, % (*n*)	16% (8/50)	0% (0/10)	15% (9/60)
Somatostatin receptor analogue, % (*n*)	6% (3/50)	20% (2/10)	8% (5/60)
Pancreatic surgery, % (*n*)	19% (12/62)	23% (3/13)	20% (15/75)

Numbers of individuals (*n*) where data is available is provided

*Mann–Whitney *U* statistical analysis, *P* > 0.05

Extra-pancreatic features, excluding neurological conditions which could be related to hypoglycaemic brain insult, were reported in nine individuals (10%) (Additional file 1: Table S4). None of these were commonly shared between patients in keeping with *HK1*-HI causing isolated pancreatic disease.

### Management and glycaemic outcome of *HK1*-HI

The median age at the last clinical update of the 90 affected individuals was 6 years (range: 2 months–66 years). Treatment details were available for 75 individuals; 60 (80%) had therapeutically managed HI, and 15 (20%) had undergone pancreatic surgery.

Of the 60 medically managed individuals, 46 (77%) had received diazoxide only (doses up to 25 mg/kg/day), and 14 (23%) had received somatostatin receptor analogues (SSRA) as an adjunct or alternative therapy to diazoxide. Eleven individuals had lost their initial response to low or moderate dose of diazoxide between the ages of 6 months and 5 years, resulting in an increased dose of diazoxide (*n* = 4), the addition of SSRA (*n* = 3), or pancreatic surgery (*n* = 4). Four further probands were reported to have remission with subsequent relapse at ages 6 weeks, 7 months, 23 years, and 50 years. Ten medically treated individuals were not receiving treatment at follow-up with medication discontinued between 3 and 49.5 years (Table 1, Additional file 1: Table S4).

The 15 individuals who underwent pancreatic surgery were diagnosed with HI in the neonatal period with surgery performed at a median age of 18 months (range: 2 weeks–32 months). Follow-up data were available on 12 individuals, which confirmed that one was without treatment, ten had ongoing hypoglycaemia requiring treatment, and one had insulin-dependent diabetes (Additional file 1: Table S4).

Twenty-two family members (9 females) with a pathogenic or likely pathogenic variant did not have a clinical diagnosis of HI at the median age of 40 years (range: 25–60 years). This included 15 obligate heterozygotes. Four individuals had reported anecdotal episodes of hypoglycaemia which had not been biochemically confirmed (Fig. 2). Survival analysis showed that by the age of 60 years, the risk of disease was 64% for family members with a *HK1* variant (Additional file 1: Fig. S3).

### Clinical characteristics of individuals with a VUS in the *HK1* regulatory region

A clinical diagnosis of HI had been made in 75% (30/40) of individuals with a VUS (24 probands and 6 family members). The median age at diagnosis was 8 months (IQR: 6–12 months) and none were born large for gestational age (Additional file 1: Table S5 and S6). The median current age of the ten unaffected family members was 33 years (range: 25–52 years).

Follow-up data were available on 29/30 affected individuals with a VUS, confirming they had been medically treated; 28 had received diazoxide only (doses up to 13.5 mg/kg/day), and one SSRA only. Seven individuals were not receiving treatment having discontinued medication between the ages of 3 and 5 years. One further proband reported a remission of HI which relapsed at the age of 7 years, after being off medication for 3 years (Additional file 1: Table S5).

## Discussion

We identified variants in the *HK1 cis*-regulatory region in 89 of 1761 probands with HI. This represents a minimum prevalence of 4% in unsolved cases based on pathogenic/likely pathogenic variants (65/1761). When variants of uncertain clinical significance are included, the prevalence rises to 5% (89/1761, all variants). This detection rate is similar to that reported in the Norwegian cohort (3%) but lower than the 9% reported by Wakeling et al. [3, 16]. This likely reflects the wider heterogeneity in our study population which included all referrals to three international genomics laboratories regardless of disease severity. These results firmly establish *HK1* as a major cause of HI with a prevalence similar, and in some cases higher, to other well-reported genetic forms of the condition including *HNF4A*-HI,* GCK*-HI, and *HADH-*HI (Additional file 1: Fig. S1) [10, 16, 32, 33]. To the best of our knowledge the findings also establish *HK1* as the most common known cause of monogenic disease due to non-coding variants affecting a *cis*-regulatory element controlling tissue-specific gene silencing [5, 6].

Twenty-three novel variants were identified in this study taking the total number of variants reported in this region to 32. Two novel SNVs allowed us to extend the originally described 42 bp minimal regulatory region by four bases (Chr10:71,108,642–71,108,687) (Fig. 1). All 46 bases within the newly defined region were disrupted by at least one indel or SNV identified in this study. This high density of variants in a non-coding *cis*-regulatory element is extremely rare and likely reflects the complex interplay between multiple transcription factors that are required for the maintenance of silencing *HK1 *in the pancreatic beta-cell during foetal development and throughout life [3]. Studies to assess the transcriptional regulation of *HK1* will be important to gain insights into mechanisms of tissue-specific gene repression and to further assess how disruption of these processes impacts on disease severity.

Our results confirm that *HK1* variants cause HI with extensive variability in clinical severity, similar to that described in *GCK*-HI [7, 9, 12]. At the most severe end of the spectrum were individuals born large for gestational age, who were diagnosed with drug-resistant HI at birth, with some still requiring high-dose medication after subtotal pancreatectomy. In contrast, some individuals had reached mid/late-adulthood without reported symptoms of hypoglycaemia. This finding of reduced penetrance is consistent with the observations of Pinney et al. who reported five asymptomatic individuals in the pedigree with linkage to *HK1* [15]. In the future it will be important to undertake clinical testing of glucose metabolism, to explore whether asymptomatic individuals show a mild form of HI, and to follow these individuals to see if symptomatic HI develops later in life as described in the 89-year-old individual reported by Pinney et al. [15].

The observation that 11 individuals had reported a loss of responsiveness to medical therapy is interesting given that for most forms of HI the severity of the disease reduces over time [34]. This finding is consistent with the observations of Henquin et al. who reported progressive loss of diazoxide efficacy in two patients in their cohort [13, 14]. For one of these, there was evidence of aberrant HK1 expression in pancreatic beta-cells, whilst in the second case a mosaic pathogenic *GCK *variant p.(Ile211Phe) was identified within the pancreas. The authors hypothesised that the diminishing responsiveness to diazoxide could result from mosaicism with the number of beta-cells with the variant increasing with ageing [13]. In our study, a de novo variant was confirmed in nine of the 11 individuals with progressive loss of responsiveness to diazoxide whilst in one proband the variant was paternally inherited and in one case inheritance could not be established.

Although this study represents the most comprehensive report to date describing *HK1* variants in HI, our work has some limitations. Our calculation of the overall prevalence of *HK1* variants may be underestimated given that individuals with the mildest forms of HI may not be referred for genetic testing, and all known genetic causes of HI were not excluded in our cohort. The number of individuals with an undetected pathogenic variant in a known HI gene is however likely to be small given that the most common genetic aetiologies had been excluded (e.g. *ABCC8* and *KCNJ11* were screened in 100% of individuals, and *GLUD1* in 79% of the cohort and 100% of individuals with reported hyperammonaemia, a feature of *GLUD1*-HI) [35]. Clinical data was also limited in patients who were lost to follow-up, a consequence of screening historic cohorts recruited over a 20-year period, this however only applied to a minority of the cohort. Furthermore, it is possible that there are slight differences in the reporting of diagnosis of HI and treatment response between clinicians. Our findings are however applicable to patients worldwide as we have studied a large number of individuals from multiple centres with different genetic ancestries, outweighing concerns over minor variations in clinician reporting. Finally, for 12 variants, there was insufficient evidence to confirm pathogenicity [22, 23]. This is due to the absence of finding a de novo occurrence of the variant which provides strong evidence for pathogenicity, incomplete penetrance of variants within families, variants residing in a region that has not yet been associated with endocrine-specific transcriptional regulation, and/or a lack of patient pancreatic tissue for *HK1* expression studies. As more reports of *HK1*-HI emerge in the literature and as the region becomes better functionally characterised, it is likely that further evidence will become available to support their pathogenicity.

## Conclusions

Through screening three large international cohorts, we have firmly established that dominant variants in the *HK1 cis*-regulatory region are a common cause of isolated HI, accounting for ~ 4–5% of genetically unsolved cases. Variants affecting the promoter region of the beta-cell disallowed gene *SLC16A1* cause exercise-induced HI with variants that disrupt distal *cis*-regulatory elements of two other genes (*PTF1A* and *FOXA2*) having also been reported to cause a monogenic disorder of insulin secretion [5, 36, 37]. Taken together these findings highlight the critical role of the non-coding genome in controlling pancreatic development and function. Through this study, we have provided a genetic diagnosis for 89 families living with HI, most of whom have waited many years to understand the cause of their disease. In doing so, we have established that *HK1*-HI is associated with variable clinical severity in terms of age at diagnosis and response to medical therapy, with some individuals remaining asymptomatic in late-adulthood. Based on these findings, we recommend that the *HK1* regulatory region is screened in all newly diagnosed children referred for HI genetic testing as well as historic cases where the genetic aetiology of HI remains unknown.

## Supplementary Information

Additional file 1: Table S1. Details of PCR primer sequences and methodology for Sanger sequencing. Table S2. Details of primer and reporter sequences and methodology for deletion screening. Table S3. Classification of *HK1* variants identified according to current guidelines. Table S4. Individual-level clinical and genetic characteristics for 90 individuals with a clinical diagnosis of congenital hyperinsulinism (HI) and a monoallelic pathogenic or likely pathogenic variant within the *HK1* regulatory region. Table S5. Individual-level clinical and genetic characteristics for 30 individuals with a clinical diagnosis of congenital hyperinsulinism (HI) and a monoallelic variant of uncertain significance within the *HK1* regulatory region. Table S6. Summary of clinical features for 30 individuals with a clinical diagnosis of congenital hyperinsulinism and a monoallelic variant of uncertain significance within the *HK1* regulatory region. Fig S1. Bar chart comparing the frequency of *HK1* variants to other known genetic causes of hyperinsulinism in the Exeter cohort (*n*=2,949). Fig S2. Pedigrees depicting inheritance of *HK1* variants of uncertain significance. Fig S3. Kaplan Meier plot showing the probability of congenital hyperinsulinism by age in individuals with a monoallelic pathogenic or likely pathogenic* HK1 *variant. 

## Data Availability

The raw sequencing data generated during the current study are not publicly available to preserve patient confidentiality. Variant call format (.vcf) files are available through collaboration to experienced teams working on approved studies examining the mechanisms, cause, diagnosis and treatment of diabetes and other beta cell disorders. Requests for collaboration will be considered by a steering committee following an application to the Genetic Beta Cell Research Bank (IRAS: 316,050, https://www.diabetesgenes.org/current-research/genetic-beta-cell-research-bank/). Contact by email should be directed to Sarah Flanagan (s.flanagan@exeter.ac.uk).

## Abbreviations

ddPCR: Droplet digital PCR
GCK: Glucokinase
HI: Hyperinsulinism
HK1: Hexokinase 1
MAF: Minor allele frequency
MLPA: Multiplex ligation-dependent probe amplification
qPCR: Quantitative PCR
SSRA: Somatostatin receptor analogue
tNGS: Targeted next-generation sequencing
VUS: Variant of uncertain significance
WGS: Whole genome sequencing
