# Supplementary material for: Non-coding cis-regulatory variants in HK1 cause congenital hyperinsulinism with variable disease severity
Source: Genome Med. 2025 Mar 3;17:17. doi: 10.1186/s13073-025-01440-w (PMC11874398; doi:10.1186/s13073-025-01440-w)
Supplement: Supplementary file 1 — Additional file 1: Table S1. Details of PCR primer sequences and methodology for Sanger sequencing. Table S2. Details of primer and reporter sequences and methodology for deletion screening. Table S3. Classification of HK1 variants identified according to current guidelines. Table S4. Individual-level clinical and genetic characteristics for 90 individuals with a clinical diagnosis of congenital hyperinsulinism (HI) and a monoallelic pathogenic or likely pathogenic variant within the HK1 regulatory region. Table S5. Individual-level clinical and genetic characteristics for 30 individuals with a clinical diagnosis of congenital hyperinsulinism (HI) and a monoallelic variant of uncertain significance within the HK1 regulatory region. Table S6. Summary of clinical features for 30 individuals with a clinical diagnosis of congenital hyperinsulinism and a monoallelic variant of uncertain significance within the HK1 regulatory region. Fig S1. Bar chart comparing the frequency of HK1 variants to other known genetic causes of hyperinsulinism in the Exeter cohort (n=2,949). Fig S2. Pedigrees depicting inheritance of HK1 variants of uncertain significance. Fig S3. Kaplan Meier plot showing the probability of congenital hyperinsulinism by age in individuals with a monoallelic pathogenic or likely pathogenic HK1 variant. [file 13073_2025_1440_MOESM1_ESM.docx]

**Additional file 1**

Table S1: Details of PCR primer sequences and methodology for Sanger sequencing...........................................2

Table S2: Details of primer and reporter sequences and methodology for deletion screening................................3

Table S3: Classification of *HK1* variants identified according to current guidelines..............................................4

Table S4: Individual-level clinical and genetic characteristics for 90 individuals with a clinical diagnosis of congenital hyperinsulinism (HI) and a monoallelic pathogenic or likely pathogenic variant within the *HK1* regulatory region......................................................................................................................................................6

Table S5: Individual-level clinical and genetic characteristics for 30 individuals with a clinical diagnosis of congenital hyperinsulinism (HI) and a monoallelic variant of uncertain significance within the *HK1* regulatory region......................................................................................................................................................................19

Table S6: Summary of clinical features for 30 individuals with a clinical diagnosis of congenital hyperinsulinism and a monoallelic variant of uncertain significance within the *HK1* regulatory region..............24

Fig S1: Bar chart comparing the frequency of *HK1* variants to other known genetic causes of hyperinsulinism in the Exeter cohort (n=2,949)...................................................................................................................................25

Fig S2: Pedigrees depicting inheritance of *HK1* variants of uncertain significance..............................................26

Fig S3: Kaplan Meier plot showing the probability of congenital hyperinsulinism by age in individuals with a monoallelic pathogenic or likely pathogenic *HK1* variant.....................................................................................27

**Table S1:** Details of PCR primer sequences and methodology for Sanger sequencing.

| **Centre** | **Primer direction** | **Sequence (5’-3’)** | **Region amplified (GRCh37/hg19)** | **Sequencer** | **Analysis software** |
| --- | --- | --- | --- | --- | --- |
| Exeter | Forward | AGCCTGGGCAACAGAAAC | chr10:71,108,536–71,108,932 (397 bp) | ABI 3730 capillary machine (Applied Biosystems) | Mutation Surveyor version 3.24 software (SoftGenetics) |
|  | Reverse | GCTACAAGCTCAGCCTCTTTC |  |  |  |
| Paris | Forward | AGCCTGGGCAACAGAAAC | chr10:71,108,536–71,108,932 (397 bp) | ABI 3730 capillary machine (Applied Biosystems) | Seqscape software version v2.6 (Applied Biosystems) |
|  | Reverse | GCTACAAGCTCAGCCTCTTTC |  |  |  |
| Magdeburg | Forward | GCCGGTGCCTGTAATCCC | chr10:71,108,434–71,108,807 (374 bp) | ABI 3500xl genetic analyzer (Life Technologies GmbH, Darmstadt, Germany) | Sequence Pilot software v.5.3.4 (JSI medical systems, Ettenheim, Germany) |
|  | Reverse | TTTCCACTGAAGCTGGG |  |  |  |

**Table S2:** Details of primer and reporter sequences and methodology for deletion screening.

| **Centre (method)** | **Description** | **Sequence (5’-3’)** | **Region covered (GRCh37/hg19)** |
| --- | --- | --- | --- |
| Exeter (TaqMan) | Forward primer | ACATCTGTCCTGTGTTTGTTTTCCA | chr10:71,108,671–71,108,776 |
|  | Reverse primer | GAAAGCTCATTAAACAACACATCCGA |  |
|  | Reporter sequence | ACTGGCAATTCTCCACTTTCT |  |
| Paris (SybrGreen) | Forward primer | AGGCAGAGTTTTTGCTTGCC | chr10:71,108,620–71,108,748 |
|  | Reverse primer | GGCTACTGGCAATTCTCCAC |  |
| Magdeburg (MLPA) | Left Probe Oligonucleotide 1 | [*1]-TCTCCTTTTCTCATTTATCCCTAAGGATA | chr10:71,107,806–71,107,863 |
|  | Right Probe Oligonucleotide 1 | TTGTAAGCACTAATTAGAGTGTAAGCTTT-[*2] |  |
|  | Left Probe Oligonucleotide 2 | [*1]-GGAGCCAACCCGAGGTAAAAGGGACTGATAG | chr10:71,109,484–71,109,545 |
|  | Right Probe Oligonucleotide 2 | GTCCCTTTTGTTATGGAGGGCAAGTGGGTAT-[*2] |  |
|  | Left Probe Oligonucleotide 3a | [*1]-GAATCAATTCATTGAGCTGGACTCAGGCAGGCATT | chr10:71,108,579–71,108,648 |
|  | Right Probe Oligonucleotide 3a | TCCCGCAGGCAGAGTTTTTGCTTGCCCTGTATTCC-[*2] |  |
|  | Left Probe Oligonucleotide 3b | [*1]-TGCCCTGTATTCCAGCACAGGCTGGTA | chr10:71,108,636–71,108,689 |
|  | Right Probe Oligonucleotide 3b | CTCGAGACACATCTGTCCTGTGTTTGT-[*2] |  |
|  | Left Probe Oligonucleotide 4 | [*1]-GTTTTCCAGTCAACGGAAGGAAAATATTTACCTTCTC | chr10:71,108,071–71,108,144 |
|  | Right Probe Oligonucleotide 4 | TTCTTGCAGCATTTGGGTGGTCTCAGTTTTCTTATAT-[*2] |  |
|  | Left Probe Oligonucleotide 5 | [*1]-CAGGTGTGAGCCACCACGCCCGGTTTAGGAACA | chr10:71,107,465–71,107,542 |
|  | Right Probe Oligonucleotide 5 | TTTAAAAAGGTCCATTCAGGTGTGGTCAGATTCTTCAGT-[*2] |  |

[*1]: GGGTTCCCTAAGGGTTGGA (left probe oligonucleotide primer binding sequence)
[*2]: TCTAGATTGGATCTTGCTGGCAC (right probe oligonucleotide primer binding sequence)

**Table S3:** Classification of *HK1* variants identified according to current guidelines.^1-3^ Variants are listed according to their genomic positions on chromosome 10 (GRCh37/hg19). *Variant reported previously.^4,5^ VUS = variant of uncertain significance.

| **Variant** | **Evidence** | **Classification** |
| --- | --- | --- |
| Deletion spanning critical region*  (Deletion breakpoints remain undetermined in 15/17 individuals) | *De novo* in 13 probands (PS2_Strong)  Pancreatic tissue staining confirmed expression of *HK1* in the beta-cells (PP4_Strong)  Variant identified in 17 probands (PS4_Moderate)  Absent from population datasets (PM2_Moderate) | Pathogenic  (2 strong,  2 moderate) |
| g.71108625_71108646delins19 | *De novo* in 1 proband (PS2_Strong)  Disrupts transcription factor binding motif (PM1_Supporting)  Absent from population datasets (PM2_Moderate) | Likely pathogenic  (1 strong,  1 moderate,  1 supporting) |
| g.71108639_71108651delins15 | Disrupts transcription factor binding motif (PM1_Supporting)  Absent from population datasets (PM2_Moderate) | VUS  (1 moderate,  1 supporting) |
| g.71108641_71108677delinsAGTAT | *De novo* in 1 proband (PS2_Strong)  Disrupts transcription factor binding motif (PM1_Supporting)  Absent from population datasets (PM2_Moderate) | Likely pathogenic  (1 strong,  1 moderate,  1 supporting) |
| g.71108642G>C | Absent from population datasets (PM2_Moderate) | VUS  (1 moderate) |
| g.71108642G>T | *De novo* in 1 proband (PS2_Strong)  Absent from population datasets (PM2_Moderate) | Likely pathogenic  (1 strong,  1 moderate) |
| g.71108642del | Absent from population datasets (PM2_Moderate) | VUS  (1 moderate) |
| g.71108645T>C* | *De novo* in 2 probands (PS2_Strong)  Variant identified in 2 probands (PS4_Supporting),  Disrupts transcription factor binding motif (PM1_Supporting)  Absent from population datasets (PM2_Moderate) | Likely pathogenic  (1 strong,  1 moderate,  2 supporting) |
| g.71108647C>G | *De novo* in 1 proband (PS2_Strong)  Disrupts transcription factor binding motif (PM1_Supporting)  Absent from population datasets (PM2_Moderate) | Likely pathogenic  (1 strong,  1 moderate,  1 supporting) |
| g.71108647C>T* | *De novo* in 2 probands (PS2_Strong)  Variant identified in 2 probands (PS4_Supporting),  Disrupts transcription factor binding motif (PM1_Supporting)  Absent from population datasets (PM2_Moderate) | Likely pathogenic  (1 strong,  1 moderate,  2 supporting) |
| g.71108647C>A | *De novo* in 1 proband (PS2_Strong)  Disrupts transcription factor binding motif (PM1_Supporting)  Absent from population datasets (PM2_Moderate) | Likely pathogenic  (1 strong,  1 moderate,  1 supporting) |
| g.71108648C>A* | *De novo* in 1 proband (PS2_Strong)  Disrupts transcription factor binding motif (PM1_Supporting)  Absent from population datasets (PM2_Moderate) | Likely pathogenic  (1 strong,  1 moderate,  1 supporting) |
| g.71108648C>G* | *De novo* in 1 proband (PS2_Strong)  Disrupts transcription factor binding motif (PM1_Supporting)  Absent from population datasets (PM2_Moderate) | Likely pathogenic  (1 strong,  1 moderate,  1 supporting) |
| g.71108648C>T* | *De novo* in 4 probands (PS2_Strong)  Pancreatic tissue staining confirmed expression of *HK1* in the beta-cells (PP4_Strong)  Variant identified in 4 probands (PS4_Moderate),  Disrupts transcription factor binding motif (PM1_Supporting)  Absent from population datasets (PM2_Moderate) | Pathogenic  (2 strong,  2 moderate,  1 supporting) |
| g.71108648del | *De novo* in 1 proband (PS2_Strong)  Disrupts transcription factor binding motif (PM1_Supporting)  Absent from population datasets (PM2_Moderate) | Likely pathogenic  (1 strong,  1 moderate,  1 supporting) |
| g.71108651_71108668del | *De novo* in 1 proband (PS2_Strong)  Disrupts transcription factor binding motif (PM1_Supporting)  Absent from population datasets (PM2_Moderate) | Likely pathogenic  (1 strong,  1 moderate,  1 supporting) |
| g.71108652A>C | Absent from population datasets (PM2_Moderate) | VUS  (1 moderate) |
| g.71108652A>G | Variant identified in 3 probands (PS4_Moderate)  Absent from population datasets (PM2_Moderate) | VUS  (2 moderate) |
| g.71108653C>T | Variant identified in 8 probands (PS4_Moderate)  Absent from population datasets (PM2_Moderate) | VUS  (2 moderate) |
| g.71108654A>C* | Variant identified in 2 probands (PS4_Supporting)  Absent from population datasets (PM2_Moderate) | VUS  (1 moderate  1 supporting) |
| g.71108654A>G | Variant identified in 2 probands (PS4_Supporting)  Absent from population datasets (PM2_Moderate) | VUS  (1 moderate  1 supporting) |
| g.71108660_71108688del | *De novo* in 1 proband (PS2_Strong)  Disrupts transcription factor binding motif (PM1_Supporting)  Absent from population datasets (PM2_Moderate) | Likely pathogenic  (1 strong,  1 moderate,  1 supporting) |
| g.71108663C>G | Disrupts transcription factor binding motif (PM1_Supporting)  Absent from population datasets (PM2_Moderate) | VUS  (1 moderate,  1 supporting) |
| g.71108663C>T | Variant identified in 3 probands (PS4_Moderate),  Disrupts transcription factor binding motif (PM1_Supporting)  Absent from population datasets (PM2_Moderate) | VUS  (2 moderate,  1 supporting) |
| g.71108664T>G | *De novo* in 1 proband (PS2_Strong)  Variant identified in 2 probands (PS4_Supporting)  Disrupts transcription factor binding motif (PM1_Supporting)  Absent from population datasets (PM2_Moderate) | Likely pathogenic  (1 strong,  1 moderate,  2 supporting) |
| g.71108665C>G* | *De novo* in 3 probands (PS2_Strong)  Variant identified in 19 probands (PS4_Moderate)  Disrupts transcription factor binding motif (PM1_Supporting)  Absent from population datasets (PM2_Moderate) | Likely pathogenic  (1 strong,  2 moderate,  1 supporting) |
| g.71108665del | *De novo* in 1 proband (PS2_Strong)  Disrupts transcription factor binding motif (PM1_Supporting)  Absent from population datasets (PM2_Moderate) | Likely pathogenic  (1 strong,  1 moderate,  1 supporting) |
| g.71108666_71108675del | Disrupts transcription factor binding motif (PM1_Supporting)  Absent from population datasets (PM2_Moderate) | VUS  (1 moderate,  1 supporting) |
| g.71108684_71108685del | *De novo* in 2 probands (PS2_Strong)  Variant identified in 3 probands (PS4_Moderate),  Disrupts transcription factor binding motif (PM1_Supporting)  Absent from population datasets (PM2_Moderate) | Likely pathogenic  (1 strong,  2 moderate  1 supporting) |
| g.71108687delT | Disrupts transcription factor binding motif (PM1_Supporting)  Absent from population datasets (PM2_Moderate) | VUS  (1 moderate,  1 supporting) |
| g.71108687T>C | *De novo* in 1 proband (PS2_Strong)  Variant identified in 2 probands (PS4_Supporting)  Disrupts transcription factor binding motif (PM1_Supporting)  Absent from population datasets (PM2_Moderate) | Likely pathogenic  (1 strong,  1 moderate,  2 supporting) |
| g.71108688_71108691del* | *De novo* in 2 probands (PS2_Strong)  Variant identified in 3 probands (PS4_Moderate)  Disrupts transcription factor binding motif (PM1_Supporting)  Absent from population datasets (PM2_Moderate) | Likely pathogenic  (1 strong,  2 moderate,  1 supporting) |

Details of how each criterion was applied to classify the *HK1* variants: PS2_Strong: Variant identified as *de novo* in at least one individual with hyperinsulinism (HI) and no family history, with maternity and paternity confirmed; PS4_Moderate: affecting a base that was disrupted in three or more unrelated individuals with HI; PS4_Supporting: affecting a base that was disrupted in two unrelated individuals with HI; PM1_Supporting: variant predicted to disrupt a binding motif of a transcription factor, perturbation of which is repeatedly shown to be pathogenic. Transcription factor binding motifs previously described in Wakeling *et al.,*^4^ code and data required to determine disrupted motifs are provided at <https://github.com/owensnick/HK1FigureNotebook.jl>; PM2_Moderate: absence of the variant in gnomAD v3,^6^ IndiGenomes,^7^ TOPMed,^8^ UK Biobank,^9^ and All of Us^10^, with the exception of g.71108653C>T with an allele count of 1/490,782 in All of Us. GnomAD SV v4.1.0 was used for the deletions spanning the critical region.^11^; PP4_Strong: Patient’s phenotype or family history is highly specific for a disease with a single genetic aetiology, used in cases where pancreatic tissue staining confirmed expression of *HK1* in the beta-cells.

**Table S4: Individual-level clinical and genetic characteristics for 90 individuals with a clinical diagnosis of congenital hyperinsulinism (HI) and a monoallelic pathogenic or likely pathogenic variant within the *HK1* regulatory region.** *****indicates individuals previously reported in Wakeling *et al.* 2022.^3^ Abbreviation: OGTT, oral glucose tolerance test.

| **Family number** | 1* | 2* | 2* | 3 | 4 | 5 | 6 |
| --- | --- | --- | --- | --- | --- | --- | --- |
| **Centre** | Exeter | Exeter | Exeter | Paris | Paris | Paris | Paris |
| **Relationship to proband** | Proband | Proband | Twin | Proband | Proband | Proband | Proband |
| **Variant**  **(inheritance)** | g.71107383–71111940del  (*de novo*) | g.71105619–71110170del  (*de novo*) | g.71105619–71110170del  (*de novo*) | Deletion encompassing critical region  (*de novo*) | Deletion encompassing critical region  (*de novo*) | Deletion encompassing critical region  (*de novo*) | Deletion encompassing critical region  (*de novo*) |
| **Sex** | Female | Male | Male | Male | Female | Female | Female |
| **Age at last clinical update (years)** | 14·9 years | 15·3 years | 15·3 years | Deceased | 20 years | 4 years | 3 years |
| **Age at clinical diagnosis of HI** | Birth | Birth | Birth | 3 days | Birth | Birth | Birth |
| **Birth weight (g), gestational age (weeks)**  **(Z-score)** | 3900, 38  (1·91) | ·· | 3645, 36  (2·07) | 3635, 40  (0·17) | ·· | 4480, 36  (4·14) | 5000, 40  (3·37) |
| **Blood glucose at diagnosis, mmol/L**  **(paired insulin, pmol/L)** | 1·2 (77) | 1·1 (1771) | 0·8 (>452) | 1·11 (170) | ·· | 0·3 (449) | 1·32 (303) |
| **Current treatment**  **(dose)** | Diazoxide  (6·5 mg/kg/d)  post-operatively | Diazoxide and  intermittent corn starch  post-operatively | Diazoxide and  intermittent corn starch  post-operatively | Diazoxide | Diazoxide  (0·9 mg/kg/d)  post-operatively | Diazoxide and Pasireotide | Diazoxide  (11 mg/kg/d) and  Octreotide  (36 mcg/kg/d) |
| **Initial response to diazoxide lost over time?** | Yes, diazoxide 5 mg/kg/d at 4 months with good response | ·· | ·· | ·· | ·· | ·· | ·· |
| **Previous**  **treatments tried** | Octreotide Sirolimusis | Diazoxide  (10 mg/kg/d) and  Lanreotide  (12·5 mg/mo) | Diazoxide  (10 mg/kg/d) and  Lanreotide  (12·5 mg/mo) | ·· | Diazoxide  (3·7-10 mg/kg/d) and Octreotide  (6-74 mcg/kg/d) | Diazoxide  (10 mg/kg/d) and  Octreotide  at 4 months of age | ·· |
| **Pancreatectomy**  **(age and year performed)** | Yes (23 months, 2008) | Yes (32 months, 2012) | Yes (32 months, 2012) | ·· | Yes (6 months) | ·· | ·· |
| **Extra-pancreatic features** | ·· | ·· | ·· | Low set ears | ·· | Dysmorphic features | Left cardiac ventricular hypertrophy |
| **Additional comments** | Active duodenitis, exocrine insufficiency | ·· | ·· | ·· | ·· | Developmental delay | Mild motor delay |

| **Family number** | 7 | 8 | 9 | 10 | 11 | 12 | 13 |
| --- | --- | --- | --- | --- | --- | --- | --- |
| **Centre** | Paris | Exeter | Exeter | Exeter | Exeter | Exeter | Exeter |
| **Relationship to proband** | Proband | Proband | Proband | Proband | Proband | Proband | Proband |
| **Variant**  **(inheritance)** | Deletion encompassing critical region  (*de novo*) | Deletion encompassing critical region  (*de novo*) | Deletion encompassing critical region  (*de novo*) | Deletion encompassing critical region  (*de novo*) | Deletion encompassing critical region  (*de* *novo*) | Deletion encompassing critical region  (*de novo*) | Deletion encompassing critical region (unknown) |
| **Sex** | Male | Male | Female | Female | Male | Female | Female |
| **Age at last clinical update (years)** | 3 years | 11·5 years | 2·3 years | 6·5 years | 0·6 years | 22·2 years | 0·7 years |
| **Age at clinical diagnosis of HI** | 2 days | 1st week | 1st week | 6 months | Birth | Birth | 2 days |
| **Birth weight (g), gestational age (weeks)**  **(Z-score)** | 4200, 36  (3·29) | 3950, 39  (1·24) | 4450, 37  (3·51) | 2700, 36  (0·26) | 3635, 40  (0·17) | 3760, 36  (2·45) | 3750, 38  (1·6) |
| **Blood glucose at diagnosis, mmol/L**  **(paired insulin, pmol/L)** | 3·3 (32)  during treatment | 2·4 (50) | ·· | 1·5 (56) | 1·1 (171) | 1·1 (··) | 1·3 (570) |
| **Current treatment**  **(dose)** | Diazoxide  (10·4 mg/kg/d) | Diazoxide | Lanreotide | Diazoxide  (13 mg/kg/d) | Diazoxide  (25 mg/kg/d) | Diazoxide  (2·5–4·5 mg/kg/d) and  Lanreotide  (90 mg/4 weeks)  post-operatively | Diazoxide |
| **Initial response to diazoxide lost over time?** | ·· | ·· | ·· | ·· | ·· | ·· | ·· |
| **Previous**  **treatments tried** | ·· | ·· | Diazoxide  (15 mg/kg/d) and  Octreotide  (30 mcg/kg/d)  at 6 months of age | ·· | ·· | Diazoxide  (up to 15 mg/kg/d) | ·· |
| **Pancreatectomy**  **(age and year performed)** | ·· | ·· | ·· | ·· | ·· | Yes (10 months, 1999) | ·· |
| **Extra-pancreatic features** | Dysmorphic features | ·· | ·· | Diplegic cerebral palsy | ·· | ·· | ·· |
| **Additional comments** | Autism spectrum disorder,  developmental delay | ·· | ·· | ·· | ·· | Epileptic seizures Haematuria/proteinuria since age 5 years | ·· |

| **Family number** | 14 | 15 | 16 | 17 | 18 | 19 | 19 |
| --- | --- | --- | --- | --- | --- | --- | --- |
| **Centre** | Exeter | Exeter | Magdeburg | Magdeburg | Exeter | Magdeburg | Magdeburg |
| **Relationship to proband** | Proband | Proband | Proband | Proband | Proband | Proband | Daughter |
| **Variant**  **(inheritance)** | Deletion encompassing critical region  (*de novo*) | Deletion encompassing critical region  (*de novo*) | Deletion encompassing critical region (unknown) | Deletion encompassing critical region (unknown) | g.71108625_ 71108646delins19  (*de novo*) | g.71108641_  71108677delinsAGTAT mosaic  (*de novo*) | g.71108641_  71108677delinsAGTAT (Paternal) |
| **Sex** | Male | Female | Female | Male | Male | Male | Female |
| **Age at last clinical update (years)** | 0·7 years | 4 years | 5 years | 18 years | 3 years | ·· | 5 years |
| **Age at clinical diagnosis of HI** | 2 days | 1 week | Birth | Birth | Birth | Birth | Birth |
| **Birth weight (g), gestational age (weeks)**  **(Z-score)** | ·· | 3740, 37  (2·05) | ·· | 4020, 40  (1·00) | 4450, 38  (2·72) | ·· | 4030, 37  (2·66) |
| **Blood glucose at diagnosis, mmol/L**  **(paired insulin, pmol/L)** | 2·05 (66) | 0·3 (72) | ·· | ·· | 1·1 (51) | ·· | 0·6 (··) |
| **Current treatment**  **(dose)** | Diazoxide and Octreotide  Insufficient response | Diazoxide  (15 mg/kg/d) | ·· | No current treatment | Diazoxide  (15 mg/kg/d)  leucine restricted diet | ·· | Diazoxide  (13 mg/kg/d) and Lanreotide  (60 mg/4 weeks) |
| **Initial response to diazoxide lost over time?** | ·· | ·· | ·· | ·· | ·· | ·· | ·· |
| **Previous**  **treatments tried** | ·· | Diazoxide  (20–25 mg/kg/day) and uncooked cornstarch | ·· | ·· | Combined diazoxide and octreotide with maximal doses, and glucagon infusion | ·· | ·· |
| **Pancreatectomy**  **(age and year performed)** | ·· | ·· | ·· | Yes (18 months) |  | Yes (6 months) | Partial pancreatectomy (2 years) |
| **Extra-pancreatic features** | ·· | ·· | ·· | ·· | ·· | ·· | ·· |
| **Additional comments** | ·· | Thalassemia,  glucose-6-phospate dehydrogenase deficiency, global developmental delay, epilepsy | Mother had pancreatectomy age 15 months | Diabetes since 11 years | ·· | ·· | ·· |

| **Family number** | 20 | 21 | 22* | 22* | 23 | 24* | 25* |
| --- | --- | --- | --- | --- | --- | --- | --- |
| **Centre** | Exeter | Exeter | Exeter | Exeter | Exeter | Exeter | Exeter |
| **Relationship to proband** | Proband | Proband | Proband | Mother | Proband | Proband | Proband |
| **Variant**  **(inheritance)** | g.71108642G>T  (*de novo*) | g.71108645T>C  (*de novo*) | g.71108645T>C (Maternal) | g.71108645T>C  (*de novo*) | g.71108647C>G  (*de novo*) | g.71108647C>T  (*de novo*) | g.71108647C>T  (*de novo*) |
| **Sex** | Female | Male | Male | Female | Male | Male | Female |
| **Age at last clinical update (years)** | 4 years | 4·5 years | 6 years | 42 years | 4 years | 12 years | 5·9 years |
| **Age at clinical diagnosis of HI** | 7 months | 9 weeks | 5 months | Neonate | 2 days | Birth | Birth |
| **Birth weight (g), gestational age (weeks)**  **(Z-score)** | 3200, 38  (0·38) | 3900, 39  (1·13) | 3800, 40  (0·53) | ·· | 3590, 39  (0·47) | 4100, 38  (1·99) | 3385, 39  (0·34) |
| **Blood glucose at diagnosis, mmol/L**  **(paired insulin, pmol/L)** | Positive glucagon stimulation test (increase of 3·8 mmol/L) | 1·3 (106) | 0·8 (13) | 2·3 (11) | 1·6 (226) | 1·4 (192) | 0·8 (315) |
| **Current treatment**  **(dose)** | No current treatment | Diazoxide  (7·2 mg/kg/d) | Diazoxide  (11·4 mg/kg/d) | No current treatment | Diazoxide  (10 mg/kg/d) | Diazoxide  (8 mg/kg/d)  post-operatively | Diazoxide  (8 mg/kg/d)  post-operatively |
| **Initial response to diazoxide lost over time?** | ·· | ·· | ·· | ·· | ·· | Initial response to 8 mg/kg/d was lost by the age of 1–2 years | Initial response lost by age 1 year |
| **Previous**  **treatments tried** | Diazoxide  (10 mg/kg/d)  until 3 years 4 months | ·· | ·· | Diazoxide  (10mg/kg/d)  until 7 years | ·· | Octreotide | Initially diazoxide  (max 25 mg/kg/d).  Diazoxide  (11 mg/kg/d) and  Lanreotide  (30 mg/28 days) |
| **Pancreatectomy**  **(age and year performed)** | ·· | ·· | ·· | ·· | ·· | Yes (2 years 1 month and 2 years 10 months, 2012) | Yes 30%  (2 years, 2018) |
| **Extra-pancreatic features** | ·· | ·· | ·· | ·· | ·· | ·· | ·· |
| **Additional comments** | ·· | ·· | ·· | ·· | No learning difficulties | Formal neurocognitive testing: abilities within normal range  (2018, age 9 years) | No learning difficulties |

| **Family number** | 26 | 27 | 28* | 29* | 30* | 31* | 32* |
| --- | --- | --- | --- | --- | --- | --- | --- |
| **Centre** | Exeter | Paris | Exeter | Exeter | Exeter | Exeter | Exeter |
| **Relationship to proband** | Proband | Proband | Proband | Proband | Proband | Proband | Proband |
| **Variant**  **(inheritance)** | g.71108647C>A  mosaic ~34%  (*de novo*) | g.71108648del  (*de novo*) | g.71108648C>T  (*de novo*) | g.71108648C>T  (*de novo*) | g.71108648C>T  (*de novo*) | g.71108648C>T  mosaic 17%  (*de novo*) | g.71108648C>A (Paternal) |
| **Sex** | Male | Male | Female | Female | Female | Female | Female |
| **Age at last clinical update (years)** | 2 months | 9 years | 9 years | 4 years | 12·4 years | 5·2 years | 4 years |
| **Age at clinical diagnosis of HI** | 2 days | 2 days | 2 weeks | 3 days | Birth | Birth | Birth |
| **Birth weight (g), gestational age (weeks)**  **(Z-score)** | ·· | 5190, 41  (3·06) | 3760, 37  (2·10) | 4000, 37  (2·60) | 2368, 36  (-0·54) | 3590, 39  (0·81) | 3910, 39  (1·50) |
| **Blood glucose at diagnosis, mmol/L**  **(paired insulin, pmol/L)** | 1·45 (5) | 0·7 (42) | 1·7 (1240) | 2·2 (151) | 2·7 (118) | 2·1 (336) | ·· |
| **Current treatment**  **(dose)** | Diazoxide  (15mg x 3/day) | Diazoxide  (4·5-6 mg/kg/d) and Octreotide  (120 mg/10 weeks)  Continuous gastrostomy feed overnight | Insulin  (9·5 U/day)  Post-operatively | Diazoxide  (15 mg/kg/d) | Diazoxide  (2·6 mg/kg/d) and  Octreotide  (16·2 mcg/kg/d) | Diazoxide  (10 mg/kg/d) and  Octreotide  (20 mcg/kg/d) | Diazoxide |
| **Initial response to diazoxide lost over time?** | Good response, but tendency to hypoglycaemia when dose outgrown | ·· | Yes, previously responsive to diazoxide | Yes, initial response to ‘low dose’ was good | ·· | Yes, lowest reported dose 9·5 mg/kg/d at 3–4 months, dose increased to 17 mg/kg/d by the age of 16 months | ·· |
| **Previous**  **treatments tried** | ·· | ·· | Diazoxide | ·· | ·· | Nifedipin | ·· |
| **Pancreatectomy**  **(age and year performed)** | ·· | ·· | Yes, 3 operations  (6-11 months) | ·· | ·· | ·· | ·· |
| **Extra-pancreatic features** | ·· | ·· | ·· | ·· | Horse-shoe kidney | ·· | ·· |
| **Additional comments** | ·· | ·· | ·· | ·· | Asthma, attention deficit hyperactivity disorder, learning disability | ·· | ·· |

| **Family number** | 32* | 33* | 34 | 35 | 36 | 37 | 37 |
| --- | --- | --- | --- | --- | --- | --- | --- |
| **Centre** | Exeter | Exeter | Exeter | Paris | Magdeburg | Magdeburg | Magdeburg |
| **Relationship to proband** | Father | Proband | Proband | Proband | Proband | Proband | Sister |
| **Variant**  **(inheritance)** | g.71108648C>A  (*de novo*) | g.71108648C>G  (*de novo*) | g.71108651_ 71108668del  mosaic 12%  (*de novo*) | g.71108660_ 71108688del  mosaic 12%  (*de novo*) | g.71108664T>G  (*de novo*) | g.71108664T>G (Paternal) | g.71108664T>G (Paternal) |
| **Sex** | Male | Female | Male | Male | Male | Female | Female |
| **Age at last clinical update (years)** | ·· | Deceased age 2·5 months (sepsis) | 7 months | 4·8 years | 4 years | 11 years | 7 years |
| **Age at clinical diagnosis of HI** | ·· | 2 weeks | 13 weeks | Birth | Birth | 9 months | Birth |
| **Birth weight (g), gestational age (weeks)**  **(Z-score)** | ·· | 3500, 40  (0·20) | 3600, 40  (0·10) | 4400, 38  (2·62) | 3780, 40  (0·49) | 3230, 40  (-0·44) | 3490, 39  (0·58) |
| **Blood glucose at diagnosis, mmol/L**  **(paired insulin, pmol/L)** | ·· | 1·6 (236) | 1·5 (493) | 2·5 (567) | 1·6 (40·3) | 1·6 (<14) | 1·7 (··) |
| **Current treatment**  **(dose)** | ·· | Octreotide  post-operatively | Diazoxide | Diazoxide  (15 mg/kg/d) | No current treatment | Diazoxide  (2·9 mg/kg/d)  Euglycaemic | Diazoxide  (4·9 mg/kg/d)  Euglycaemic |
| **Initial response to diazoxide lost over time?** | ·· | ·· | ·· | Initially good response to ‘low dose’, then off diazoxide for 4 weeks, restarted at 6 weeks of age with good response. Increased to 15 mg/kg/d by 3 years of age | ·· | ·· | ·· |
| **Previous**  **treatments tried** | ·· | ·· | Octreotide  (7 mcg/kg/d)  for 3 days | ·· | ·· | ·· | ·· |
| **Pancreatectomy**  **(age and year performed)** | Yes (2 weeks, 2011) | Yes (3 weeks, 2019) | ·· | ·· | ·· | ·· | ·· |
| **Extra-pancreatic features** | ·· | Microcephaly, hepatosplenomegaly | ·· | ·· | ·· | ·· | ·· |
| **Additional comments** | ·· | ·· | ·· | ·· | ·· | ·· | ·· |

| **Family number** | 37 | 38 | 38 | 38 | 38 | 38 | 38 |
| --- | --- | --- | --- | --- | --- | --- | --- |
| **Centre** | Magdeburg | Paris | Paris | Paris | Paris | Paris | Paris |
| **Relationship to proband** | Father | Proband | Mother | First cousin | Uncle | First cousin once removed | First cousin once removed |
| **Variant**  **(inheritance)** | g.71108664T>G (Unknown) | g.71108665C>G (Maternal) | g.71108665C>G (Presumed paternal) | g.71108665C>G (Paternal) | g.71108665C>G (Presumed paternal) | g.71108665C>G (Paternal) | g.71108665C>G (Paternal) |
| **Sex** | Male | Male | Female | Female | Male | Female | Female |
| **Age at last clinical update (years)** | ·· | 31 years | 50 years | 50 years | 78 years | 6·4 years | 19 years |
| **Age at clinical diagnosis of HI** | ·· | 2 years | ·· | 12 years | ·· | 5 months | 19 years |
| **Birth weight (g), gestational age (weeks)**  **(Z-score)** | ·· | ·· | ·· | ·· | ·· | 3380, 38  (0·79) | ·· |
| **Blood glucose at diagnosis, mmol/L**  **(paired insulin, pmol/L)** | ·· | ·· | ·· | ·· | ·· | 0·8 (97) | ·· |
| **Current treatment**  **(dose)** | ·· | Lanreotide | ·· | No current treatment | No medical treatment but needs to split meals | Lanreotide  (only partial control) | ·· |
| **Initial response to diazoxide lost over time?** | ·· | ·· | ·· | ·· | ·· | Yes, by age 3 years | ·· |
| **Previous**  **treatments tried** | ·· | ·· | ·· | Previously diazoxide responsive stopped due to side effects. Treated with Lanreotide until 49·5 years old | ·· | Diazoxide with initial good response | ·· |
| **Pancreatectomy**  **(age and year performed)** | ·· | ·· | ·· | ·· | ·· | ·· | ·· |
| **Extra-pancreatic features** | ·· | ·· | ·· | ·· | ·· | Hearing loss | ·· |
| **Additional comments** | ·· | ·· | ·· | ·· | ·· | Speech delay. Normal growth and development | Father feels tired before meals |

| **Family number** | 38 | 38 | 38 | 38 | 39* | 40 | 41* |
| --- | --- | --- | --- | --- | --- | --- | --- |
| **Centre** | Paris | Paris | Paris | Paris | Exeter | Exeter | Exeter |
| **Relationship to proband** | Second cousin once removed | Second cousin once removed | Second cousin once removed | Second cousin | Proband | Proband | Proband |
| **Variant**  **(inheritance)** | g.71108665C>G (Maternal) | g.71108665C>G (Maternal) | g.71108665C>G (Maternal) | g.71108665C>G (Presumed paternal) | g.71108665C>G  (*de novo*) | g.71108665C>G (Paternal) | g.71108665C>G  (*de novo*) |
| **Sex** | Female | Male | Female | Female | Male | Female | Male |
| **Age at last clinical update (years)** | 30 years | 36 years | 31 years | 60 years | 18 years | 12·5 years | 5 years |
| **Age at clinical diagnosis of HI** | ·· | ·· | ·· | ·· | 2 days (resolved)  7 months (relapse) | 13 months | 3 months |
| **Birth weight (g), gestational age (weeks)**  **(Z-score)** | ·· | ·· | ·· | ·· | 3700, 42  (-0·10) | 4000, 40  (1·32) | 1810, 37  (-2·66) |
| **Blood glucose at diagnosis, mmol/L**  **(paired insulin, pmol/L)** | ·· | ·· | ·· | ·· | 1·4 (112) | 1·5 (13·2) | 1·6 (60) |
| **Current treatment**  **(dose)** | ·· | ·· | ·· | ·· | Octreotide LAR | No current treatment | Diazoxide  (15 mg/kg/d) |
| **Initial response to diazoxide lost over time?** | ·· | ·· | ·· | ·· | ·· | ·· | ·· |
| **Previous**  **treatments tried** | ·· | ·· | ·· | ·· | Diazoxide until 15 years with only partial response  (poor compliance) | Diazoxide until 12·5 years | Somatostatin prior to referral for 15 days |
| **Pancreatectomy**  **(age and year performed)** | ·· | ·· | ·· | ·· | ·· | ·· | ·· |
| **Extra-pancreatic features** | ·· | ·· | ·· | ·· | ·· | ·· | ·· |
| **Additional comments** | ·· | ·· | ·· | ·· | Epilepsy | Seizures (relapsed after diazoxide started). Father had impaired fasting glucose and impaired glucose tolerance observed on OGTT once at different time points further OGTTs (N=8) were normal | ·· |

| **Family number** | 42 | 43 | 44 | 44 | 45 | 45 | 45 |
| --- | --- | --- | --- | --- | --- | --- | --- |
| **Centre** | Exeter | Exeter | Paris | Paris | Paris | Paris | Paris |
| **Relationship to proband** | Proband | Proband | Proband | Second cousin | Proband | Mother | Second cousin |
| **Variant**  **(inheritance)** | g.71108665C>G (Unknown) | g.71108665C>G  (*de novo*) | g.71108665C>G (Presumed paternal) | g.71108665C>G (Presumed maternal) | g.71108665C>G (Maternal) | g.71108665C>G (Presumed maternal) | g.71108665C>G (Presumed maternal) |
| **Sex** | Male | Female | Male | Male | Male | Female | Male |
| **Age at last clinical update (years)** | 16·5 years | 3·3 years | 8·5 years | 3 years | 9·1 years | ·· | 33 years |
| **Age at clinical diagnosis of HI** | 17 months | 18 months | 10 months | 8 months | 11 months | ·· | 6 months |
| **Birth weight (g), gestational age (weeks)**  **(Z-score)** | 3287, 40  (-0·59) | 2730, 38  (-0·72) | 2730, 39  (-1·45) | 3800, 41  (0·18) | 3050, 39  (-0·73) | ·· | 3500, 40  (-0·12) |
| **Blood glucose at diagnosis, mmol/L**  **(paired insulin, pmol/L)** | 1·9 (146) | 1·5 (88) | 1·4 (··) | 2·8 (106) | 1·3 (257) | 2·5 (··) | ·· |
| **Current treatment**  **(dose)** | Diazoxide  (0·64 mg/kg/d) | Diazoxide  (6·5 mg/kg/d) | Diazoxide  (8·4 mg/kg/d) | Diazoxide  (15 mg/kg/d) | Diazoxide  (1·8 mg/kg/d) | No current treatment | No current treatment |
| **Initial response to diazoxide lost over time?** | ·· | ·· | ·· | ·· | ·· | ·· | ·· |
| **Previous**  **treatments tried** | ·· | ·· | ·· | ·· | ·· | ·· | Diazoxide as a child |
| **Pancreatectomy**  **(age and year performed)** | ·· | ·· | ·· | ·· | ·· | ·· | ·· |
| **Extra-pancreatic features** | ·· | ·· | ·· | ·· | ·· | ·· | ·· |
| **Additional comments** | Presented with seizures but no further seizures | ·· | Mild motor delay. Father has imperious need to eat | ·· | ·· | Hypoglycaemia 1 hour after a heavy meal | ·· |

| **Family number** | 46 | 47 | 47 | 48 | 48 | 48 | 48 |
| --- | --- | --- | --- | --- | --- | --- | --- |
| **Centre** | Paris | Paris | Paris | Paris | Paris | Paris | Paris |
| **Relationship to proband** | Proband | Proband | Third cousin | Proband | First cousin | First cousin | Uncle |
| **Variant**  **(inheritance)** | g.71108665C>G (Maternal) | g.71108665C>G (Maternal) | g.71108665C>G (Paternal) | g.71108665C>G (Presumed maternal) | g.71108665C>G (Presumed paternal) | g.71108665C>G (Presumed paternal) | g.71108665C>G  Affected  obligate carrier  (Unknown) |
| **Sex** | Male | Male | Male | Female | Male | Female | Male |
| **Age at last clinical update (years)** | 4 years | 15 years | 2 years | 52 years | 34 years | 35·5 years | ·· |
| **Age at clinical diagnosis of HI** | 6 months | 11 months | 21 months | 26 years | 8 months | 20 years |  |
| **Birth weight (g), gestational age (weeks)**  **(Z-score)** | 3070, 39  (-0·68) | 3100, 38  (-0·17) | 3470, 41  (-0·54) | 2400, 41  (-3·03) | ·· | ·· | ·· |
| **Blood glucose at diagnosis, mmol/L**  **(paired insulin, pmol/L)** | 0·32 (81) | ·· | ·· | 2·2 (49) | ·· | 1·9 (··) | ·· |
| **Current treatment**  **(dose)** | Diazoxide  (6 mg/kg/d) | Diazoxide  (25 mg x 3/d) | ·· | Diazoxide  (0·78 mg/kg/d) | Diazoxide  (5·6 mg/kg/d) | Diazoxide  (0·43 mg/kg/d) | Diazoxide  (2 mg/kg/d) |
| **Initial response to diazoxide lost over time?** | ·· | ·· | ·· | ·· | ·· | ·· | ·· |
| **Previous**  **treatments tried** | ·· | Diazoxide  (2·6 mg/kg/d)  at age 7 years | ·· | Started diazoxide at age 45 years.  Initially 1·17 mg/kg/d | ·· | Patient stopped therapy (Diazoxide 1·6 mg/kg/d) at 45 years old. This caused hypoglycaemia that was managed with feeding. Treatment was resumed at age 35·5 | ·· |
| **Pancreatectomy**  **(age and year performed)** | ·· | ·· | ·· | ·· | ·· | ·· | ·· |
| **Extra-pancreatic features** | ·· | ·· | ·· | ·· | ·· | ·· | ·· |
| **Additional comments** | Several seizures | ·· | ·· | ·· | ·· | Daughter had seizures when she was 5 years old | ·· |

| **Family number** | 49 | 50 | 50 | 51 | 51 | 52 | 53 |
| --- | --- | --- | --- | --- | --- | --- | --- |
| **Centre** | Paris | Paris | Paris | Paris | Paris | Paris | Paris |
| **Relationship to proband** | Proband | Proband | Father | Proband | Sister | Proband | Proband |
| **Variant**  **(inheritance)** | g.71108665C>G (Unknown) | g.71108665C>G (Paternal) | g.71108665C>G (Unknown) | g.71108665C>G (Paternal) | g.71108665C>G (Paternal) | g.71108665C>G (Maternal) | g.71108665C>G (Paternal) |
| **Sex** | Male | Male | Male | Female | Female | Male | Female |
| **Age at last clinical update (years)** | 36 years | 12·5 years | 43 years | 3 years | 0.7 years | 7·5 years | 4 years |
| **Age at clinical diagnosis of HI** | 13 months | 19 months | ·· | 2 years | 8 months | 4 months | 19 months |
| **Birth weight (g), gestational age (weeks)**  **(Z-score)** | 3770, 42  (0·03) | 3380, 40  (-0·39) | 3610, 40  (0·12) | 3390, 39  (0·35) | ·· | 3270, 38  (0·20) | 3560, 39  (0·74) |
| **Blood glucose at diagnosis, mmol/L**  **(paired insulin, pmol/L)** | Diagnosis: 1·2  Relapse: 2·2 (35) | 2·6 (576) | 1·9 (20) | 2·3 (20) | ·· | ·· | 1·4 (80·6) |
| **Current treatment**  **(dose)** | No current treatment | Diazoxide  (4·8 mg/kg/d) | No current treatment | Diazoxide  (2·7 mg/kg/d) | ·· | No current treatment | No current treatment |
| **Initial response to diazoxide lost over time?** | ·· | ·· | ·· | ·· | ·· | ·· | ·· |
| **Previous**  **treatments tried** | Diazoxide until age 14 years | ·· | ·· | ·· | ·· | Diazoxide previously | Diazoxide stopped  age 3 years |
| **Pancreatectomy**  **(age and year performed)** | ·· | ·· | ·· | ·· | ·· | ·· | ·· |
| **Extra-pancreatic features** | ·· | ·· | ·· | ·· | ·· | ·· | ·· |
| **Additional comments** | Relapse age 23 years | ·· | Several episodes of syncope during adolescence.  Normal glucose metabolism at 43 years (fasting blood glucose, insulin levels, HbA1c, and OGTT) | Father had several episodes of post-prandial faintness and fatigue after fasting. Paternal grandfather had hypoglycaemia as a child | ·· | Speech delay | ·· |

| **Family number** | 53 | 54 | 55 | 56 | 57 | 58 | 59 |
| --- | --- | --- | --- | --- | --- | --- | --- |
| **Centre** | Paris | Paris | Paris | Magdeburg | Exeter | Exeter | Exeter |
| **Relationship to proband** | Brother | Proband | Proband | Proband | Proband | Proband | Proband |
| **Variant**  **(inheritance)** | g.71108665C>G (Paternal) | g.71108665C>G (Unknown) | g.71108665C>G (Unknown) | g.71108665C>G (Unknown) | g.71108665del  (*de* *novo*) | g.71108684_ 71108685del (Unknown) | g.71108684_ 71108685del  (*de novo*) |
| **Sex** | Male | Male | Male | Female | Female | Male | Male |
| **Age at last clinical update (years)** | 8·5 years | 66 years | 5 years | ·· | 5 years | ·· | 8·7 years |
| **Age at clinical diagnosis of HI** | 2 years | 8 years | 18 months | Birth | 5 months | 1 day | 8 weeks |
| **Birth weight (g), gestational age (weeks)**  **(Z-score)** | 3230, 37  (0·60) | ·· | 1880, 35  (-1·48) | 2800, 40  (-1·51) | 3700, 40  (0·66) | 3500, 39  (0·27) | 3620, 39  (0·53) |
| **Blood glucose at diagnosis, mmol/L**  **(paired insulin, pmol/L)** | 2·7 (16·7) | 1·4 (253) | ·· | 1 (··) | ·· (83·3) | 3·1 (417) | 2·4 (164) |
| **Current treatment**  **(dose)** | No current treatment | Diazoxide  (10 mg/kg/d)  Insufficient response | No current treatment | No current treatment | Diazoxide  (15 mg/kg/d) | ·· | Diazoxide  (4·5 mg/kg/d) |
| **Initial response to diazoxide lost over time?** | ·· | ·· | ·· | ·· | Initial good response to 5 mg/kg/d  (age 11 months).  Increased to 15 mg/kg/d by age 5 years, had hypoglycaemic seizures | ·· | ·· |
| **Previous**  **treatments tried** | Diazoxide stopped  age 4 years | Diazoxide during childhood, no treatment for 30 years. Relapse age 50 years with  Occasional hypoglycaemia. | Diazoxide stopped  age 3·5 years | ·· | ·· | ·· | Corn starch 1·7 g/kg at night |
| **Pancreatectomy**  **(age and year performed)** | ·· | ·· | ·· | ·· | ·· | Yes | ·· |
| **Extra-pancreatic features** | ·· | ·· | ·· | ·· | ·· | ·· | ·· |
| **Additional comments** | ·· | Absence seizures since age 3 years | Mother had transient neonatal hypoglycaemia. Maternal grandmother had hypoglyceamia during pregnancy at age 20 and pancreatectomy | Euglycaemic since age 3 years | Developmental delay, seizures | ·· | Long-term feeding problems, slow resolution |

| **Family number** | 60 | 61 | 62 | 63* | 64 | 65 |
| --- | --- | --- | --- | --- | --- | --- |
| **Centre** | Exeter | Exeter | Exeter | Exeter | Exeter | Exeter |
| **Relationship to proband** | Proband | Proband | Proband | Proband | Proband | Proband |
| **Variant**  **(inheritance)** | g.71108684_ 71108685del  (*de novo*) | g.71108687T>C (Unknown) | g.71108687T>C  mosaic 27%  (*de novo*) | g.71108688_  71108691del  mosaic 30%  (*de novo*) | g.71108688_ 71108691del  (unknown) | g.71108688_ 71108691del  (*de novo*) |
| **Sex** | Female | Male | Male | Male | Female | Female |
| **Age at last clinical update (years)** | 1·6 years | 6 years | 3·1 years | 6 years | 3 years | 1·8 years |
| **Age at clinical diagnosis of HI** | 8 weeks | 1 day | 1^st^ week | Birth | 2 days | 2 days |
| **Birth weight (g), gestational age (weeks)**  **(Z-score)** | 2900, 40  (-1·25) | 4155, 38  (2·10) | 3500, 40  (-0·12) | 3490, 39  (0·25) | 4190, 38  (2·51) | 4040, 37  (2·68) |
| **Blood glucose at diagnosis, mmol/L**  **(paired insulin, pmol/L)** | 1·4 (240) | 1·1 (261) | 1·7 (150) | 2·0 (163) | 1·4 (50) | 2·3 (90·3) |
| **Current treatment**  **(dose)** | Diazoxide  (3·8 mg/kg/d) | Diazoxide  (13 mg/kg/d) | Diazoxide with  no response | Diazoxide  (13 mg/kg/d) and  Octreotide LAR  (30mg 3 weekly) | Diazoxide with  partial response | Diazoxide  (12·5 mg/kg/d) and  Octreotide LAR  (20 mg/month)  Not fully responsive |
| **Initial response to diazoxide lost over time?** | ·· | ·· | ·· | Yes, initially responsive, unresponsive age 6 months, dose increased, Octreotide added at age 12 months | ·· | ·· |
| **Previous**  **treatments tried** | Glucagon  (max dose 20 mcg/kg/hr) | ·· | Octreotide, Nifedipin, Diazoxide | ·· | Octreotide injections age 3 months | ·· |
| **Pancreatectomy**  **(age and year performed)** | ·· | ·· | Yes  (between 2018–2021) | ·· | ·· | ·· |
| **Extra-pancreatic features** | ·· | ·· | ·· | ·· | Depressed nasal bridge, right Erbs palsy, B/L vitreous opacity, pulmonary stenosis | ·· |
| **Additional comments** | Seizures at presentation, no developmental delay | Ataxia hypotonia in new-born period. Gastrostomy at 7 months. Specific language disorder | Developmental delay, seizures | ·· | ·· | Gastrostomy inserted at 8 months of age |

**Table S5: Individual-level clinical and genetic characteristics for 30 individuals with a clinical diagnosis of congenital hyperinsulinism (HI) and a monoallelic variant of uncertain significance within the *HK1* regulatory region.**

| **Family number** | 66 | 67 | 68 | 68 | 69 | 70 | 71 |
| --- | --- | --- | --- | --- | --- | --- | --- |
| **Centre** | Exeter | Exeter | Paris | Paris | Exeter | Exeter | Exeter |
| **Relationship to proband** | Proband | Proband | Proband | Mother | Proband | Proband | Proband |
| **Variant**  **(inheritance)** | g.71108639_ 71108651delins15 (Unknown) | g.71108642G>C  (Unknown) | g.71108642del (Maternal) | g.71108642del (Unknown) | g.71108652A>C (Unknown) | g.71108652A>G (Unknown) | g.71108652A>G (Unknown) |
| **Sex** | Male | Male | Female | Female | Female | Female | Female |
| **Age at last clinical update (years)** | 11·2 years | 13 years | 3 years | 27 years | 3·1 years | 1·2 years | 3·4 years |
| **Age at clinical diagnosis of HI** | 8 months | 11 months | 11 months | 7 months | Birth | 9 months | 8 months |
| **Birth weight (g), gestational age (weeks)**  **(Z-score)** | 3000, 40  (-1·24) | 3710, 39  (0·73) | 3430, 39  (0·45) | 4020, 40  (1·37) | 2800, 38  (-0·55) | 2600, 38  (-1·04) | 3200, 39  (-0·08) |
| **Blood glucose at diagnosis, mmol/L**  **(paired insulin, pmol/L)** | 2·2 (65) | 1·9 (111) | 1·87 (181) | ·· | ·· | 2·5 (30·6) | 1·7 (16·7) |
| **Current treatment**  **(dose)** | Diazoxide | Diazoxide  (4·1 mg/kg/d) | Diazoxide  (7 mg/kg/d) | No current treatment | Diazoxide | Diazoxide | Diazoxide  (8·4 mg/kg/d) |
| **Initial response to diazoxide lost over time?** | ·· | ·· | ·· | ·· | ·· | ·· | ·· |
| **Previous**  **treatments tried** | ·· | ·· | ·· | Diazoxide until  age 3 years 7 months | ·· | ·· | ·· |
| **Extra-pancreatic features** | ·· | Grade 4 unilateral vesico-ureteric reflux,  Aortic root dilatation* | ·· | ·· | ·· | ·· | ·· |
| **Additional comments** | ·· | Anxiety, autism spectrum disorder level 2, attention deficit hyperactivity disorder. Developmental delay. Mild intellectual disability (Full-scale intelligence quotient 62) Difficulty eating and maintaining weight* | ·· | Partial epilepsy | ·· | ·· | Vineland Adaptive Behavior Scales III recorded moderately low scores in general level of ability, language, Daily Living Skills and Socialisation with Motor skills at a Low range. Short duration seizures |

**The additional features observed in this individual are considered likely to be co-incidental, resulting from a second confirmed genetic condition in the child.*

| **Family number** | 72 | 72 | 73 | 74 | 75 | 76 | 77 |
| --- | --- | --- | --- | --- | --- | --- | --- |
| **Centre** | Exeter | Exeter | Exeter | Exeter | Exeter | Exeter | Paris |
| **Relationship to proband** | Proband | Monozygotic twin | Proband | Proband | Proband | Proband | Proband |
| **Variant**  **(inheritance)** | g.71108652A>G (Maternal) | g.71108652A>G (Maternal) | g.71108653C>T (Paternal) | g.71108653C>T (Paternal) | g.71108653C>T (Maternal) | g.71108653C>T (Maternal) | g.71108653C>T (Maternal) |
| **Sex** | Male | Male | Female | Male | Male | Female | Male |
| **Age at last clinical update (years)** | 9 years | 9 years | 11 months | 1·9 years | 4 months | 8·3 years | 12 years |
| **Age at clinical diagnosis of HI** | 6 months | 6 months | 4 months | 4 weeks | 4 months | 2 years 5 months | 5 months |
| **Birth weight (g), gestational age (weeks)**  **(Z-score)** | 2200, 36  (-1·23) | 2000, 36  (-1·70) | 2700, 36  (-1·30) | ·· | 2150, 38  (-2·34) | 2750, 37  (-0·16) | 3450, 38  (0·60) |
| **Blood glucose at diagnosis, mmol/L**  **(paired insulin, pmol/L)** | 2·5 (41·7) | 2·7 (40·3) | 1·2 (129) | 3·7 (15·3)  whilst on treatment | 2·1 (33·47) | 1·9 (36·8) | 3·4 (792) |
| **Current treatment**  **(dose)** | No current treatment | No current treatment | Diazoxide  (10 mg/kg/d) | Diazoxide | Diazoxide | Diazoxide  (2 mg/kg/d) re-started age 7 years | No current treatment |
| **Initial response to diazoxide lost over time?** | ·· | ·· | ·· | ·· | ·· | ·· | ·· |
| **Previous**  **treatments tried** | Diazoxide with max dose of 10 mg/kg/d, until 4 years of age | Diazoxide with max dose of 10 mg/kg/d, until 5 years of age | ·· | ·· | ·· | Diazoxide (8 mg/kg/d) for 2 years. 3 years of no treatment, normoglycaemia | Diazoxide  (8-10 mg/kg/d)  stopped age 9 years |
| **Extra-pancreatic features** | ·· | ·· | Cortical blindness | Bilateral profound sensorineural deafness | ·· | ·· | ·· |
| **Additional comments** | Epilepsy, developmental delay  Twin birth | Absence seizures, developmental delay  Twin birth | Seizures, developmental delay | Developmental delay | Twin birth, tonic clonic convulsion. Ataxic gait mainly truncal. Global developmental delay, Epilepsy | Seizures since 9 months | Seizures, developmental delay. Mother had childhood epilepsy and hypoglycaemia symptoms with hypoglycaemia confirmed once during non-diabetic pregnancy |

| **Family number** | 77 | 78 | 79 | 80 | 81 | 82 | 83 |
| --- | --- | --- | --- | --- | --- | --- | --- |
| **Centre** | Paris | Paris | Paris | Paris | Exeter | Exeter | Paris |
| **Relationship to proband** | Brother | Proband | Proband | Proband | Proband | Proband | Proband |
| **Variant**  **(inheritance)** | g.71108653C>T (Maternal) | g.71108653C>T (Unknown) | g.71108653C>T (Unknown) | g.71108653C>T (Unknown) | g.71108654A>C (Unknown) | g.71108654A>G (Paternal) | g.71108654A>G (Paternal) |
| **Sex** | Male | Male | Male | Male | Female | Male | Male |
| **Age at last clinical update (years)** | 6 years | 6 years | 5·8 years | 1·6 years | 6·5 years | 1·9 years | 3·6 years |
| **Age at clinical diagnosis of HI** | 6 months | 9 months | 8 months | 11 months | 3 months | 1 year | 1 year |
| **Birth weight (g), gestational age (weeks)**  **(Z-score)** | 3620, 38  (0·96) | 3300, 37  (0·75) | 3270, 41  (-0·98) | 3890, 38  (1·54) | 3100, 37  (0·65) | 2800, 38  (-0·84) | 3050, 38  (-0·28) |
| **Blood glucose at diagnosis, mmol/L**  **(paired insulin, pmol/L)** | ·· | 1·72 (51) | 1·3 (31) | 2·3 (43·8) | 1·1 (53·8) | 2·3 (264) | 2 (62) |
| **Current treatment**  **(dose)** | Diazoxide  (3·9 mg/kg/d) | No current treatment | No current treatment | Diazoxide  (9 mg/kg/d) | No current treatment | Diazoxide | Diazoxide  (108 mg/kg/d) |
| **Initial response to diazoxide lost over time?** | ·· | ·· | ·· | ·· | ·· | ·· | ·· |
| **Previous**  **treatments tried** | ·· | Age 3 years, Diazoxide  (4·8 mg/kg/d) | Diazoxide  (3·7 mg/kg/d-  4·2 mg/kg/d)  until age 3 years | ·· | Diazoxide  (13·5 mg/kg/d)  stopped age 6 years | ·· | ·· |
| **Extra-pancreatic features** | ·· | ·· | ·· | ·· | ·· | ·· | ·· |
| **Additional comments** | ·· | Father's cousin has HI, diagnosed age 17 months, diazoxide treated until age 10 | Adrenal insufficiency. Paternal great-aunt had hypoglycaemia as a child | ·· | Epilepsy | Developmental delay | ·· |

| **Family number** | 83 | 84 | 85 | 86 | 87 | 87 | 87 |
| --- | --- | --- | --- | --- | --- | --- | --- |
| **Centre** | Paris | Exeter | Paris | Paris | Paris | Paris | Paris |
| **Relationship to proband** | Sister | Proband | Proband | Proband | Proband | Father | Brother |
| **Variant**  **(inheritance)** | g.71108654A>G (Paternal) | g.71108663C>G (Paternal) | g.71108663C>T (Paternal) | g.71108663C>T  (Unknown) | g.71108663C>T (Paternal) | g.71108663C>T (Unknown) | g.71108663C>T (Paternal) |
| **Sex** | Female | Male | Male | Male | Female | Male | Male |
| **Age at last clinical update (years)** | 1·2 years | 8 years | 9 years | 2 years | 21 years | 48 years | 25 years |
| **Age at clinical diagnosis of HI** | 11 months | 7 months | 8 years | 18 months | 12 years | 43 years | 25 years |
| **Birth weight (g), gestational age (weeks)**  **(Z-score)** | 3250, 39  (-0·28) | 3000, 38  (-0·39) | 3270, 38  (0·20) | 4300, 40  (1·60) | 2820, 40  (-1·51) | 3350, 40  (-0·45) | ·· |
| **Blood glucose at diagnosis, mmol/L**  **(paired insulin, pmol/L)** | ·· | ·· (840) | 2·1 (44) | 1·83 (··) | 1·7 (22) | 2·6 (85·9) | 2·4 (··) |
| **Current treatment**  **(dose)** | Diazoxide  (10 mg/kg/d) | Diazoxide  (1·2 mg/kg/d) | Diazoxide  (5 mg/kg/d) | Diazoxide | Diazoxide  (4 mg/kg/d) | Diazoxide  (2·7 mg/kg/d) | ·· |
| **Initial response to diazoxide lost over time?** | ·· | ·· | ·· | ·· | ·· | ·· | ·· |
| **Previous**  **treatments tried** | ·· | Diazoxide  (10 mg/kg/d) | ·· | ·· | ·· | ·· | ·· |
| **Extra-pancreatic features** | ·· | ·· | ·· | ·· | ·· | ·· | ·· |
| **Additional comments** | ·· | Symptomatic since 3 days old | Hunger cravings since birth | ·· | ·· | Grandfather allegedly ate sugar all day | ·· |

| **Family number** | 88 | 89 |
| --- | --- | --- |
| **Centre** | Exeter | Exeter |
| **Relationship to proband** | Proband | Proband |
| **Variant**  **(inheritance)** | g.71108666_  71108675del  (Unknown) | g.71108687delT (Unknown) |
| **Sex** | Female | Female |
| **Age at last clinical update (years)** | ·· | 23·3 years |
| **Age at clinical diagnosis of HI** | 8 months | 1 day |
| **Birth weight (g), gestational age (weeks)**  **(Z-score)** | 2519, 36  (-0·17) | 3170, 37  (0·81) |
| **Blood glucose at diagnosis, mmol/L**  **(paired insulin, pmol/L)** | 2 (131) | 2·5 (281) |
| **Current treatment**  **(dose)** | Diazoxide | Lanreotide  with recurrent hypoglycaemic episodes |
| **Initial response to diazoxide lost over time?** | ·· | ·· |
| **Previous**  **treatments tried** | ·· | Previously only partially responsive to diazoxide |
| **Extra-pancreatic features** | ·· | ·· |
| **Additional comments** | ·· | ·· |

**Table S6:** Summary of clinical features for 30 individuals with a clinical diagnosis of congenital hyperinsulinism and a monoallelic variant of uncertain significance within the *HK1* regulatory region.

|  | **Summary data (n=30)** |
| --- | --- |
| Median age at follow-up in years (range) | 6 (0·3–48) |
| Female sex, % (data available on n=30) | 40% (n=12) |
| Median age at diagnosis in days, [IQR] (data available on n=30) | 243 [182–358] |
| Median glucose at presentation, mmol/L (paired insulin, pmol/L)  (data available on n=23) | 2 (53·8) |
| Median birth weight Z-score, [IQR] (data available on n=28) | -0·23 [-1·00–0·67] |
| Medical management, % (data available on n=29) | 100% (n=29)  n=28 diazoxide, (of which 7 were off medication at follow-up)  n=1 lanreotide |
| Pancreatic surgery, % (data available on n=29) | 0% (n=0) |


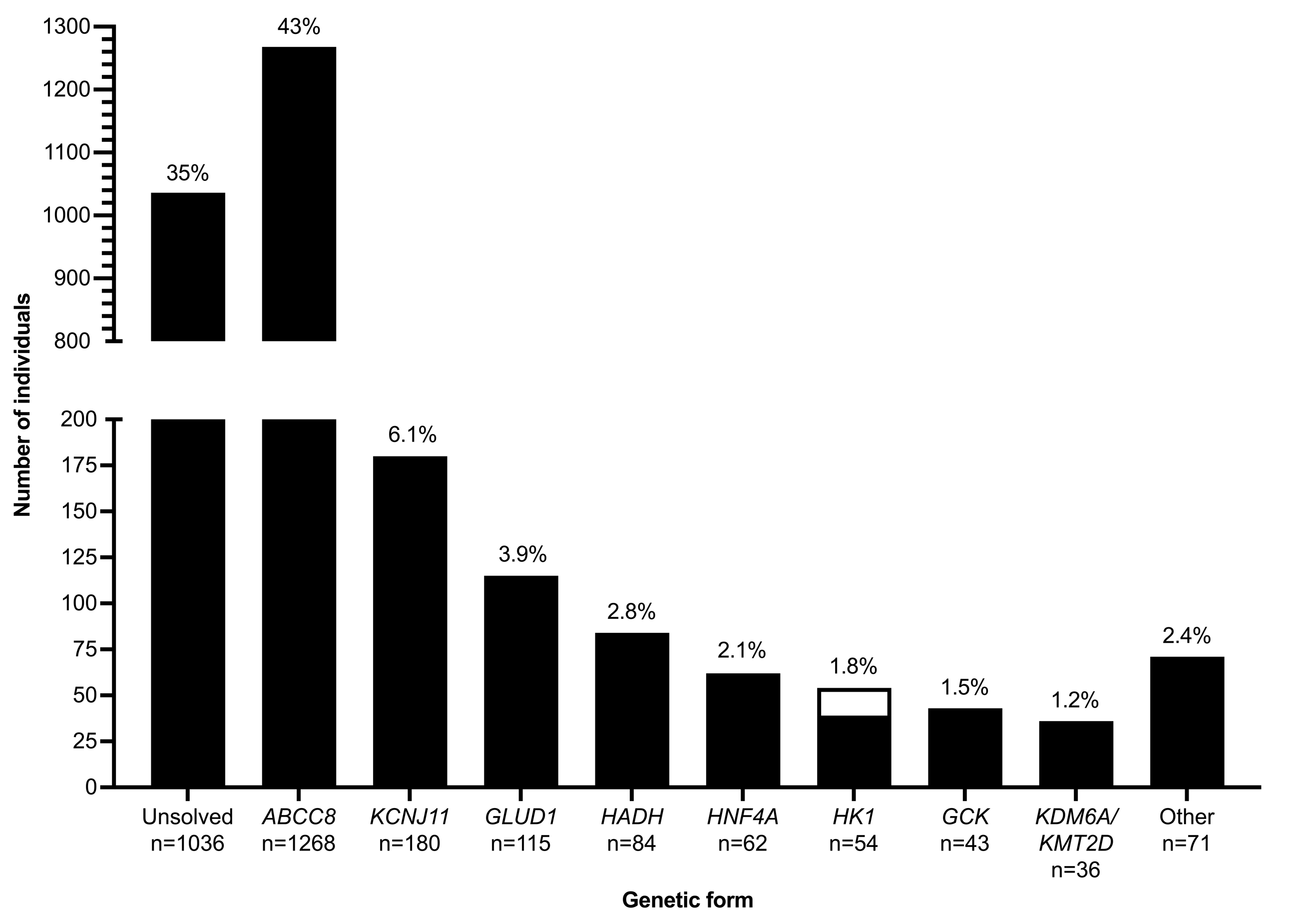


**Fig S1: Bar chart comparing the frequency of *HK1* variants to other known genetic causes of hyperinsulinism in the Exeter cohort (n=2,949). “**Other” includes: *EP300, NSD1, FOXA2, CACNA1D, PMM2, HNF1A, INSR* and large CNVs. The bar for *HK1* includes all variants identified in this study, with the portion represented by the non-filled bar indicating the variants currently classified as being of uncertain clinical significance.

**
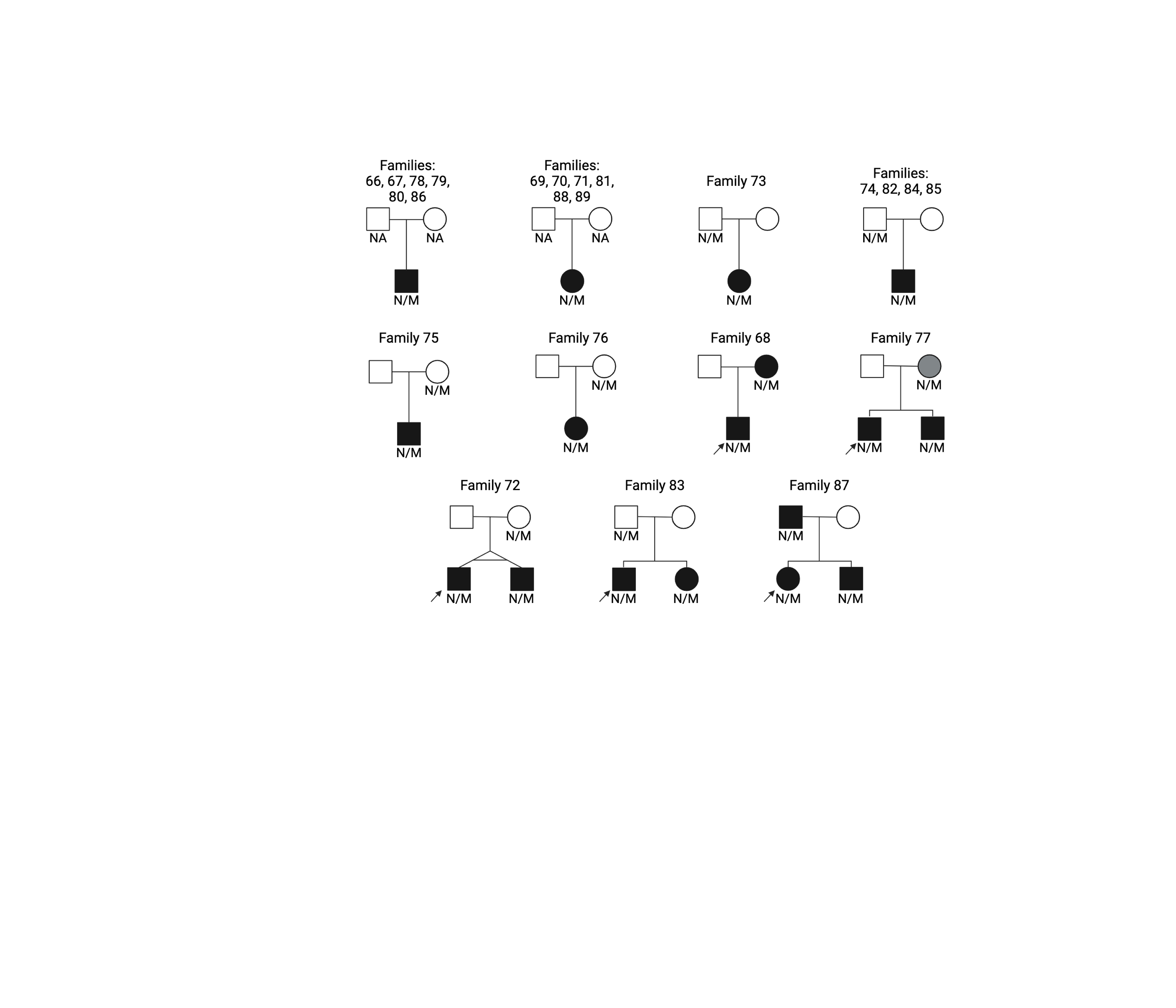
**

**Fig S2: Pedigrees depicting inheritance of *HK1* variants of uncertain significance (n=24).** Squares, males; circles, females; diamonds, unknown sex; filled black symbols, clinical diagnosis of congenital hyperinsulinism; filled grey symbols, anecdotal evidence of hypoglycaemia; M, *HK1* variant; N, no variant; NA, DNA not available. Arrows indicate probands in larger pedigrees.


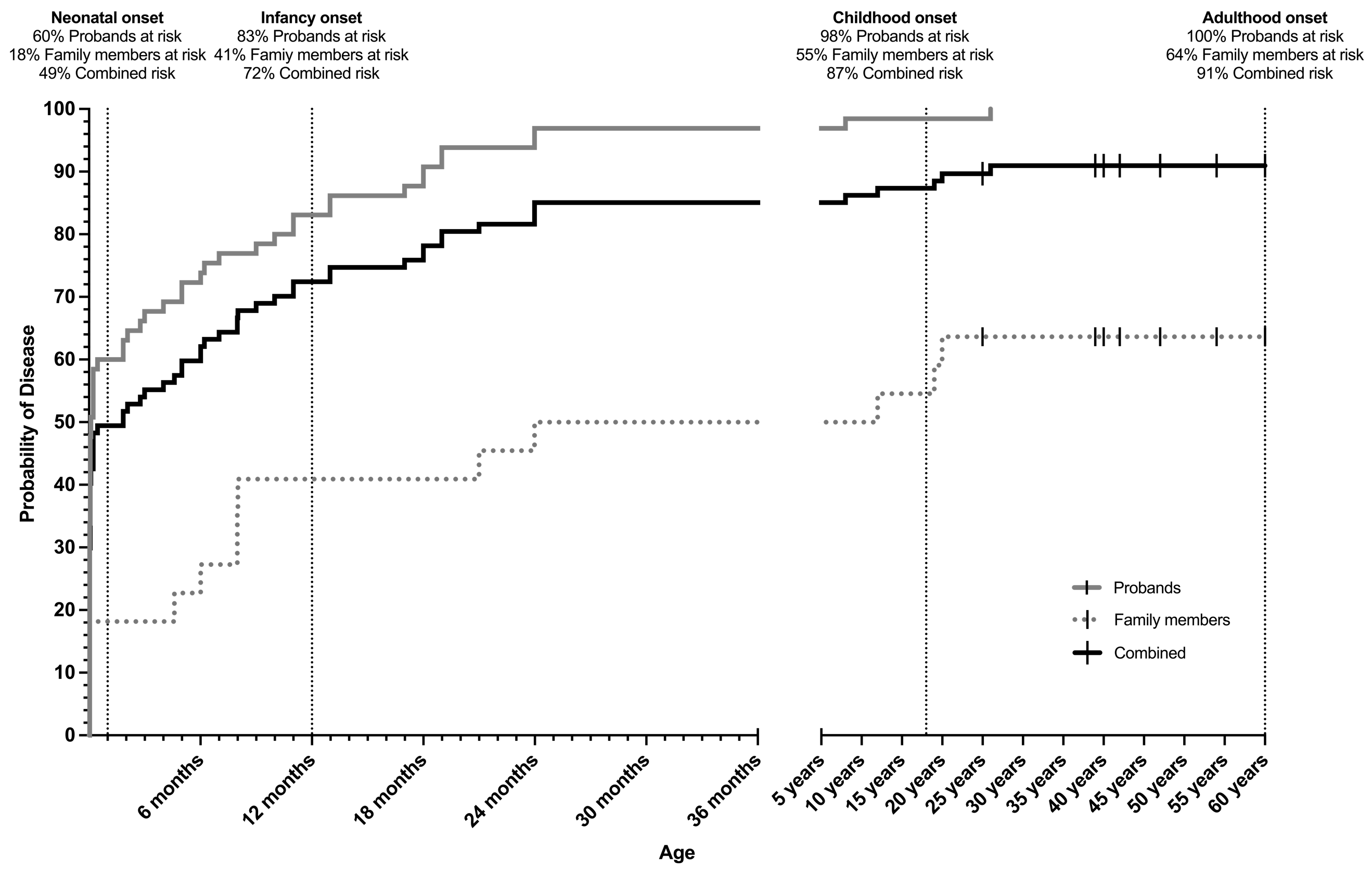


**Fig S3: Kaplan Meier plot showing the probability of congenital hyperinsulinism by age in individuals with a monoallelic pathogenic or likely pathogenic *HK1* variant.** Data available for 87/112 individuals. The solid grey line shows the probability of disease for probands (n=65), the grey dashed line shows the probability of disease for family members (n=22) and the solid black line shows the combined probability of disease (n=87). Dashes indicate censored points.

**References:**

1. Ellingford JM, Ahn JW, Bagnall RD, Baralle D, Barton S, Campbell C, et al. Recommendations for clinical interpretation of variants found in non-coding regions of the genome. Genome Med. 2022;14(1):73.

2. Richards S, Aziz N, Bale S, Bick D, Das S, Gastier-Foster J, et al. Standards and guidelines for the interpretation of sequence variants: a joint consensus recommendation of the American College of Medical Genetics and Genomics and the Association for Molecular Pathology. Genet Med. 2015;17(5):405-24.

3. Durkie M, Cassidy E-J, Berry I, Owens M, Turnbull C, Scott RH, et al. ACGS Best Practice Guidelines for Variant Classification in Rare. 2023.

4. Wakeling MN, Owens NDL, Hopkinson JR, Johnson MB, Houghton JAL, Dastamani A, et al. Non-coding variants disrupting a tissue-specific regulatory element in HK1 cause congenital hyperinsulinism. Nature Genetics. 2022;54(11):1615-20.

5. Velde CD, Molnes J, Berland S, Njølstad PR, Molven A. Clinical and genetic characteristics of congenital hyperinsulinism in Norway: A nationwide cohort study. J Clin Endocrinol Metab. 2024.

6. Chen S, Francioli LC, Goodrich JK, Collins RL, Kanai M, Wang Q, et al. A genomic mutational constraint map using variation in 76,156 human genomes. Nature. 2024;625(7993):92-100.

7. Jain A, Bhoyar RC, Pandhare K, Mishra A, Sharma D, Imran M, et al. IndiGenomes: a comprehensive resource of genetic variants from over 1000 Indian genomes. Nucleic Acids Research. 2020;49(D1):D1225-D32.

8. Taliun D, Harris DN, Kessler MD, Carlson J, Szpiech ZA, Torres R, et al. Sequencing of 53,831 diverse genomes from the NHLBI TOPMed Program. Nature. 2021;590(7845):290-9.

9. Sudlow C, Gallacher J, Allen N, Beral V, Burton P, Danesh J, et al. UK Biobank: An Open Access Resource for Identifying the Causes of a Wide Range of Complex Diseases of Middle and Old Age. PLOS Medicine. 2015;12(3):e1001779.

10. Denny JC, Rutter JL, Goldstein DB, Philippakis A, Smoller JW, Jenkins G, et al. The "All of Us" Research Program. N Engl J Med. 2019;381(7):668-76.

11. Collins RL, Brand H, Karczewski KJ, Zhao X, Alföldi J, Francioli LC, et al. A structural variation reference for medical and population genetics. Nature. 2020;581(7809):444-51.
